# Aminosteroids from Buxaceae and Apocynaceae Plants as Antiprotozoal Agents: Structural Diversity and Structure–Activity Relationships

**DOI:** 10.3390/ijms27219999

**Published:** 2026-11-08

**Authors:** Justus W. Mukavi, Lizanne Schäfer, Lara U. Szabó, Charles O. Nnadi, Julia B. Althaus, Pascal Mäser, Thomas J. Schmidt

**Affiliations:** 1 University of Münster, Institute of Pharmaceutical Biology and Phytochemistry, PharmaCampus, Corrensstrasse 48, D-48149 Münster, Germany; justusmukavi32@gmail.com (J.W.M.); lizanne.schaefer@gmail.com (L.S.); laraszabo@web.de (L.U.S.); charles.nnadi@unn.edu.ng (C.O.N.); julia.althaus@uni-muenster.de (J.B.A.); 2 Swiss Tropical and Public Health Institute (Swiss TPH), Kreuzstrasse 2, CH-4123 Allschwil, Switzerland; pascal.maeser@swisstph.ch; 3 University of Basel, Petersplatz 1, CH-4003 Basel, Switzerland

**Keywords:** aminosteroid alkaloid, Apocynaceae, Buxaceae, *Holarrhena*, *Buxus*, *Pachysandra*, antiprotozoal activity, *Trypanosoma brucei rhodesiense*, *Plasmodium falciparum*, cytotoxicity, 3D QSAR

## Abstract

As part of our search for new natural chemicals with bioactivity against protozoan pathogens, we have previously reported on the isolation, identification and antiprotozoal activity of over 100 alkaloids with an aminosteroid or related scaffold. We have now investigated the hypothesis that these chemically related compounds also share common mechanisms of action regarding their activity against *Trypanosoma brucei rhodesiense* (human East African trypanosomiasis) and *Plasmodium falciparum* (tropical malaria). Furthermore, their cytotoxic activity against mammalian cells (L6 rat skeletal myoblasts) was also included in the investigation. We found that mammalian cytotoxicity is generally lower and there is only a low degree of mutual correlation between the susceptibility of the two parasites and mammalian cells to the compounds’ toxic effects. With the molecular targets being unknown, the 3D-QSAR study was conducted using the Open3Dqsar software. The resulting models show the main structural features influencing the different biological effects. They indicate that indeed related molecular targets underly the cytotoxic, antitrypanosomal and antiplasmodial activity based on structural determinants sufficiently different enough to warrant a significant level of parasite-selectivity. The results of this study offer structural explanations for the observed differences in aminosteroids’ action against the mentioned pathogens and provide a starting point for optimization and simplification of the natural structures towards leads for antiprotozoal drug development.

## 1. Introduction

Diseases caused by unicellular eukaryotic (“protozoan”) parasites such as *Plasmodium*, *Trypanosoma* and *Leishmania* species remain a major challenge to human health and wellbeing [1,2]. The identification of new chemical entities with antiprotozoal bioactivity is an important goal since new leads for the development of drugs against Malaria, Trypanosomiasis and Leishmaniasis are needed in light of increasing parasite resistance, toxicity/unwanted effects and other shortcomings of existing therapies [3,4]. Natural products such as quinine and artemisinin have been the basis of antiprotozoal chemotherapy [3] and still represent a vast repository of potentially active drug leads to be discovered [5]. In our group we have discovered that aminosteroids (AS) from plants of the families Apocynaceae and Buxaceae often possess strong and selective bioactivity against malaria parasites (*Plasmodium falciparum*) and African trypanosomes (*Trypanosoma brucei* and others, responsible for Human and Animal African Trypanosomiases) [6,7,8,9,10,11]. In our quest for new antiprotozoal natural products, we have so far isolated 100 AS alkaloids from five plant species, namely, *Buxus sempervirens* [6,7], *B. obtusifolia* [8], *Pachysandra terminalis* [9] (Buxaceae) and from *Holarrhena africana* (a synonym of *H. floribunda*) [10] as well as *H. pubescens* [11] (Apocynaceae). They were tested in vitro for their activity against the protozoan parasites *Trypanosoma brucei rhodesiense* (*Tbr*; human African trypanosomiasis, HAT), *Plasmodium falciparum* (*Pf*; tropical malaria) and for cytotoxicity against mammalian cells (L6 rat skeletal myoblasts) [6,7,8,9,10,11]. An investigation on the antitrypanosomal mechanism of action (MoA) of some AS from *H. africana* have indicated that they target the parasite’s single mitochondrion, where they cause a breakdown of the mitochondrial membrane potential and a decrease in ATP concentration; an arrest of the cell cycle at the G2 phase was also observed [12]. Due to the high degree of structural similarity, it is straightforward to assume that common MoA and structure–activity relationships (SARs) underly the antiparasitic activity of these compounds. An earlier study has already shown the main structure–activity relationships for antiprotozoal and cytotoxic activity for the aminosteroids from *H. africana* [13]. In the present work, we have now investigated the antiplasmodial and antitrypanosomal SARs for the overall set of aminosteroids from both plant families.

**Figure 1:**
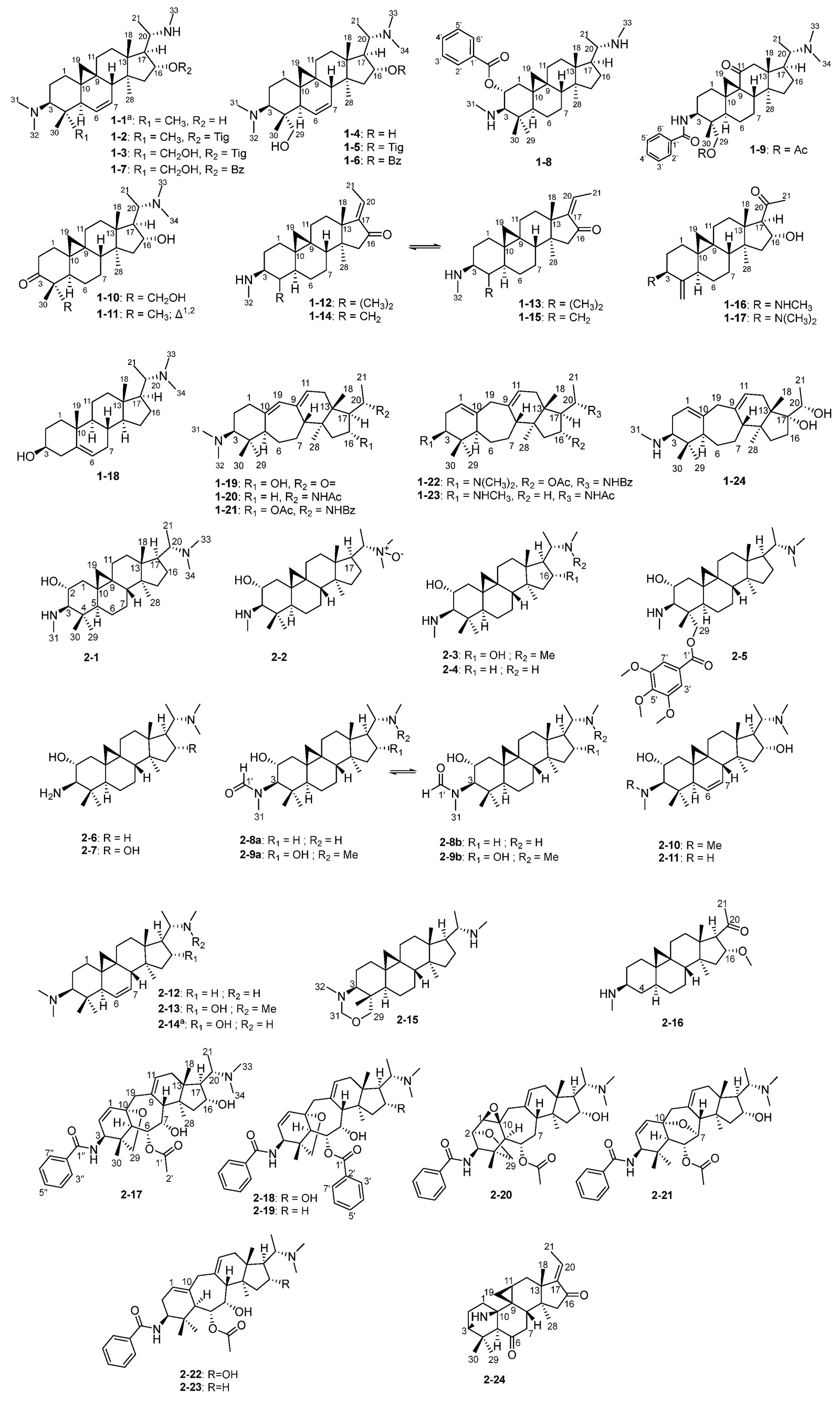

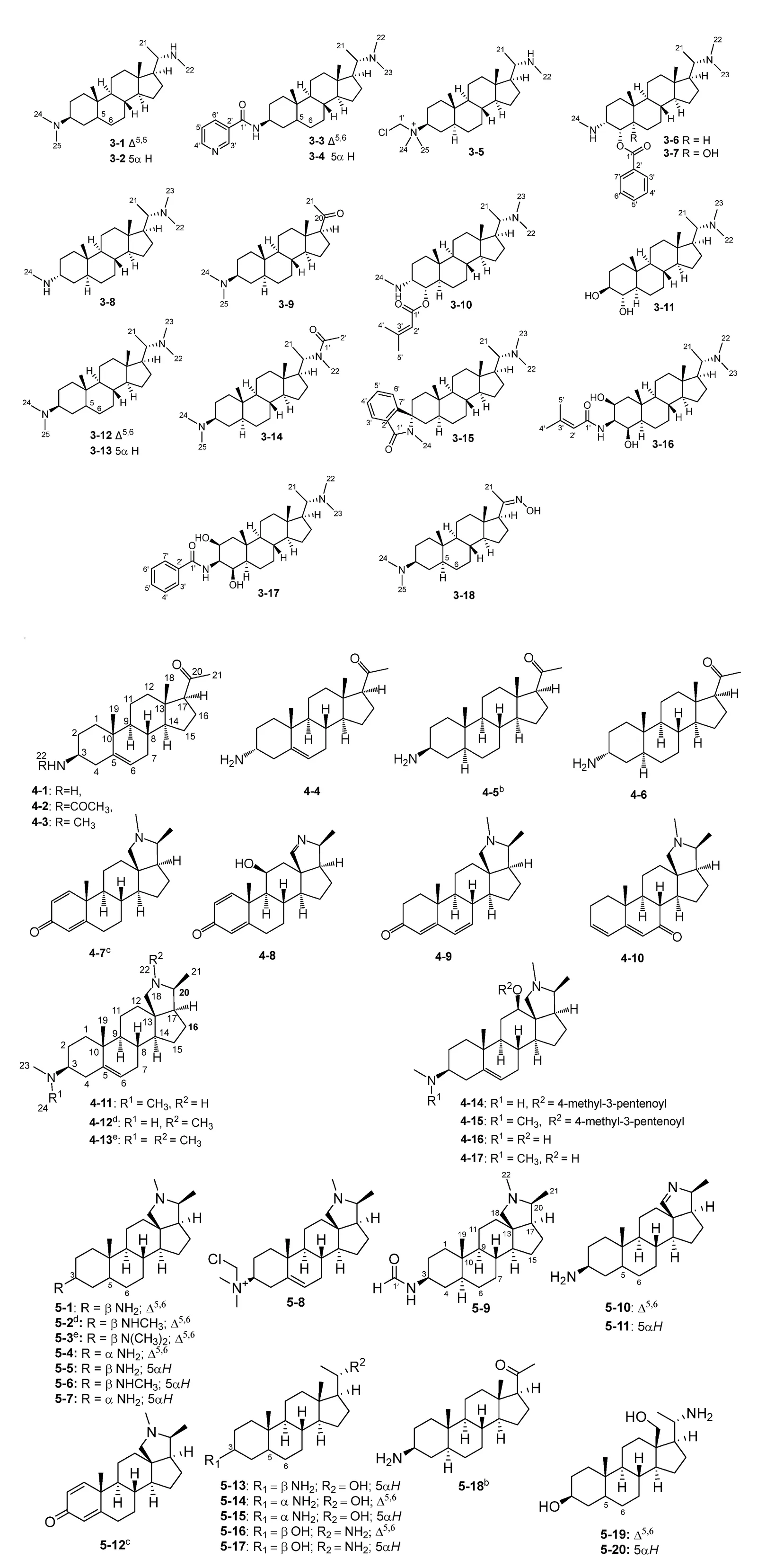
Chemical structures of the compounds under investigation. Structures are numbered by their plants of origin. **1-1**–**1-24**: *Buxus sempervirens* [6,7], **2-1**–**2-24**: *B. obtusifolia* [8], **3-1–3-18**: *Pachysandra terminalis* [9]; **4-1–4-17**: *Holarrhena floribunda* (Syn. *H. africana*) [10]; **5-1–5-20**: *H. pubescens* [11]. For individual bioactivity data, please see Supplementary Materials, Table S1. ^a–e^ Five compounds isolated independently from two different plants and shown twice, the pairs of identical structures being labeled with the same superscript letter (a–e). The mean biological activity values (pIC_50_) of these independent isolates were used in the QSAR analyses.

The chemical structures of the compounds under study are shown in Figure 1 and their bioactivity data are summarized in Table S1 (Supplementary Materials).

## 2. Results

### 2.1. Structural Diversity of the Investigated Compounds

As becomes evident from Figure 1, the chemical structures of the compounds investigated in this study possess either a regular sterane or a 9(10 → 19)*abeo*-sterane ring system. The former occurs in compounds of the typical nor-cycloartanoid *Buxus*-alkaloids with their characteristic cyclopropane moiety comprising C-9, C-10 and C-19 (compounds with a 9b,19 cyclosteroid core: **1-1**–**1-17** and **2-1**–**2-16**) [7,8] or in the form of regular steroids (i.e., pregnane skeleton) obtained from *Pachysandra terminalis* (**3-1**–**3-18**) [9] and the two *Holarrhena* species (**4-1**–**4-17** and **5-1**–**5-20**) [10,11]. The 9(10 → 19)*abeo*-sterane skeleton of compounds **1-19**–**1-24** and **2-17**–**2-23** is derived from the 9b,19 cyclosteroid (nor-cycloartane) system by cleavage of the bond between C-9 and C-10 thus forming a 7-membered ring instead of the cyclopropanocyclohexane partial structure. One novel compound isolated from *B. obtusifolia* [8], **2-24** (obtusiaminocyclin) is a special case, namely, an unprecedented 11b, 19-cyclosteroid bearing a cyclopropane moiety including C-9, C-19 and C-11 fused to a sterane tetracycle. Thus, all compounds under study share rather similar isoprenoid scaffolds, however with quite diverse substituents at various positions. On one hand, the overall similarity in the core structure indicates that the compounds may share a common mechanism of action and molecular target(s), while, on the other hand, the variable substituent decoration should then be held responsible for the different levels of bioactivity observed (see below). With respect to substitution pattern, most compounds share a nitrogen substituent at C-3 which may either be an amino or an amide group, the latter hosting various carboxylic acid moieties ranging from formamide via senecio amide to benzamide and nicotinamide groups. It is noteworthy that congeners bearing an oxygen-substituent at C-3 (which is biosynthetically simpler and far more common among the natural triterpenes and steroids as a whole) appear to be a rare exception in these plants. The side chain attached to C-17 in many cases contains a second nitrogen, typically attached to C-20 and in many cases occurring as an amino but also occasionally as amide group. This latter nitrogen in the particular subgroup of *Holarrhena* alkaloids of the conanine/conessine type (**4-7**–**4-17** and **5-1**–**5-12**) forms an additional five-membered ring.

Some compounds with unusual structural features for the current series may deserve special mentioning, such as, e.g., the *N*_20_-oxide **2-2**, the *N*_20_-oxime **3-18**, the morpholine-derivative **2-15**, the spiro-amide **3-15** or the unusual 4,4-bis-demethyl cycloartanoid sterane **2-16**, which may represent a biogenetic intermediate between the 9,19-cyclosteroids and pregnane series.

### 2.2. Activity–Activity Relationships of the Investigated Compounds

**Figure 2:**
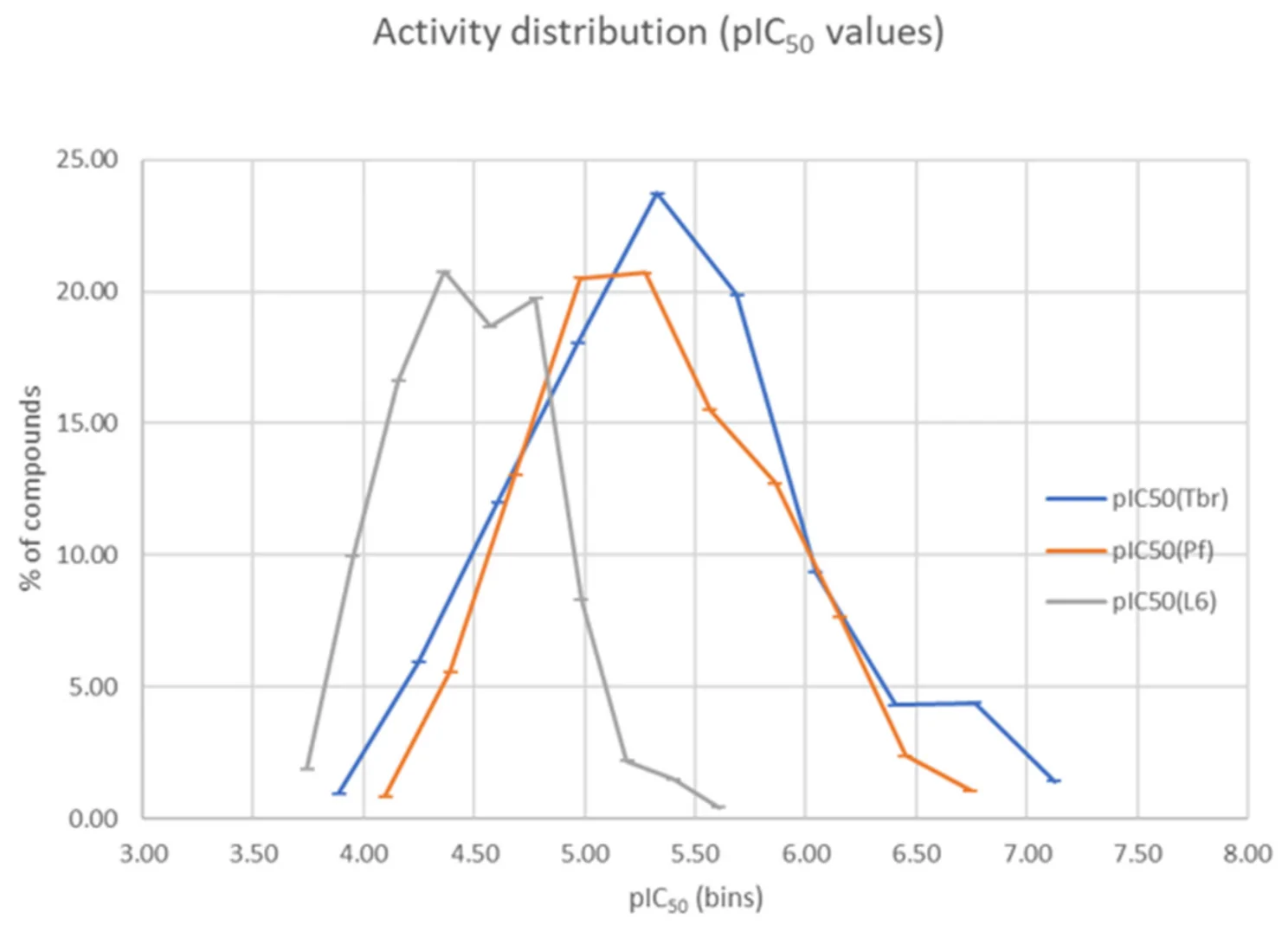
Distribution of bioactivity data. In each set, the compounds were grouped in 10 bioactivity bins (tick marks) between their minimum and maximum pIC_50_ values. The percentage of compounds in each bin was plotted against pIC_50_.

The distribution of bioactivity data (−log10 IC_50_ (mol/L) = pIC_50_ values) under study, namely, antiprotozoal activity against *Tbr* and *Pf* as well as cytotoxicity against L6 cells of all tested compounds, was analyzed and found to resemble normal distribution (Figure 2). It is important to note that the averages of the (wanted) antiprotozoal activities were about one log10 unit higher than that of the (unwanted) mammalian cytotoxicity reflecting an overall significant parasite-selectivity.

**Figure 3:**
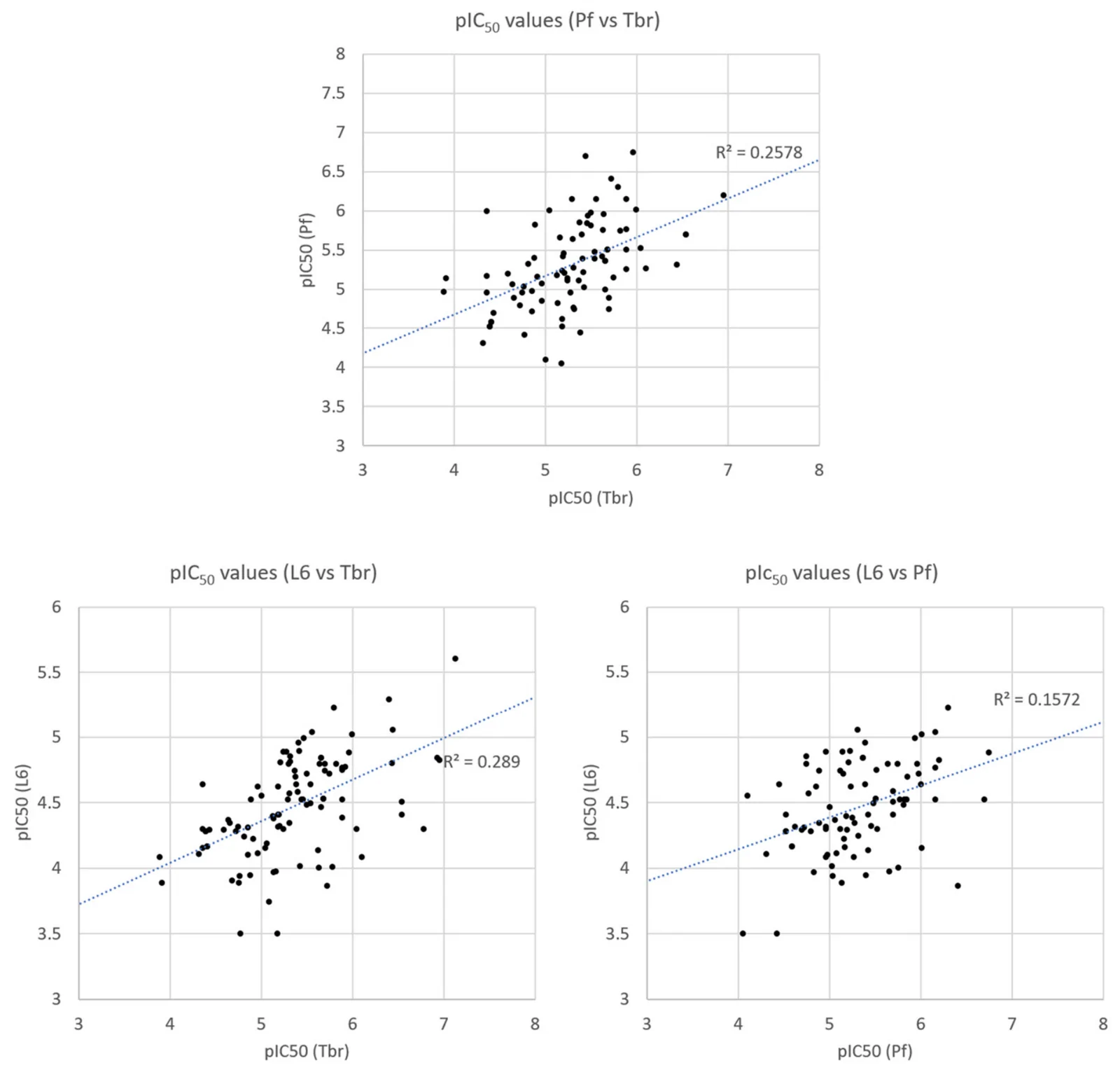
Pairwise scatterplots of the investigated bioactivities with linear trendlines and coefficients of determination.

If the observed biological activity against the different organisms/cells were due to identical mechanisms of action, i.e., an identical molecular target in each of the organisms, a significant degree of mutual correlation between the activity data should be expected. Therefore, scatterplots of the pIC_50_ values of the two antiparasitic and cytotoxic activity were generated (Figure 3) and the mutual correlations investigated. As becomes apparent from these plots, the degree of linear relationship between the activities is very low, indicating that—even if there were related molecular targets and MoA against these cells—they are sufficiently different to warrant a good basis of significant selectivity.

### 2.3. ADME Properties of the Investigated Compounds

To assess the overall physicochemical properties relevant for the absorption, distribution, metabolism and excretion (ADME) of the investigated set of aminosteroids and their potential drug- or leadlikeness, all structures were submitted in the form of their SMILES string representations to the SwissADME web tool [14] and analyzed. Table 1 gives an overview of the resulting computed/predicted properties and their distribution within the dataset. The full set of predicted ADME data is reported in the Supplementary Materials, Tables S1–S3. From the summary in Table 1 it becomes evident that the investigated series of 100 compounds in general has rather favorable properties for potential leads to orally available drugs: Only few compounds (10%) fail the Lipinski rule of 5 [15] and only 9 are predicted to be non-GI absorbable. Most are predicted to be soluble and to have good bioavailability. With respect to potential application against *T. brucei* which affects the central nervous system, it is noteworthy that the majority (72%) of the compounds are predicted to permeate the blood–brain barrier. As shown by the BOILED-egg representation (Figure S1, Supplementary Materials), ref. [16] the vast majority of the compounds under study are likely to be absorbed after oral administration by passive diffusion and to penetrate the blood–brain barrier, which would be particularly advantageous for drugs against parasites residing in the central nervous system (CNS) such as *Tbr* in the encephalic stage. As another advantage, only relatively few of the compounds Are predicted to be inhibitors of five major cytochrome P450 (CYP) enzymes so that little interference with these main players of human phase I metabolism is to be expected. However, about half of the compounds are predicted to be substrates of the cellular efflux pump, P-glycoprotein (Pgp). This might—in spite of good passive permeability—present an obstacle for these compounds’ absorption and their permeation into the CNS, since Pgp is expressed in gut mucosa and in the capillary endothelial cells forming the BBB. In summary, many of the compounds are likely to have a favorable predicted ADME profile and high potential drug- or leadlikeness.

### 2.4. Structure–Activity Relationships

In some of our previous communications on the compounds under study, we have already gained insights into structure–activity relationships with the smaller datasets then available. Particularly, 3D quantitative structure–activity relationship (3D QSAR) models were established for the anti-*Tbr* and cytotoxic activity of the compounds obtained from *H. floribunda* [13]. As major conclusions drawn from this study on the *Holarrhena* alkaloids, an amino group attached to position 3 of the steroid skeleton was found essential for high antitrypanosomal activity while closure of an additional pyrrole ring with the nitrogen atom connecting C-18 and C-20 leading to the conanine-type alkaloids appeared to cause a dramatic increase of selectivity (antitrypanosomal >> cytotoxic activity) over the compounds with a simpler tetracyclic pregnane core [13]. For the then relatively small dataset (17 compounds), it was quite easy to obtain clear-cut and statistically sound 3D QSAR models using the comparative molecular field analysis (CoMFA) [17] approach implemented in the Open3DQSAR software [18]. Moreover, it was possible to use these 3D QSAR models to make correct predictions of the antitrypanosomal activity for two *Buxus* alkaloids available to us at that time [13]. Having acquired so many more compounds from Buxaceae and also from another *Holarrhena* species, it was thus a very interesting (but demanding) task to perform a greatly extended 3D QSAR study with the present dataset of much larger structural diversity and to include also the antiplasmodial activity against *Pf*, for which no QSAR model of aminosteroids was previously reported. Considering the structural similarity between the compounds under study, it was straightforward to conceive the working hypothesis that all or most of these aminosteroids share a common MoA and one or more common target(s) in each of the investigated organisms/cell types, so that common SARs for each of the parasites and for cytotoxicity of the whole dataset or at least most of its representatives were likely to exist.

To investigate this hypothesis, the Open3DQSAR software (v. 2.281 [18]) was used in combination with the Molecular Operations Environment (MOE, rel. 2024.06) [19]. 3D molecular models for each compound were generated and optimized with the latter. The initial manual alignment of the molecular models as applied by Nnadi et al. [13] was improved by using the flexible alignment option in MOE, by which a coherent alignment could be obtained for all 100 structures in the dataset (Supplementary Materials, Figure S2). The aligned molecular models were then exported as sdf files, along with the respective bioactivity data (pIC_50_ values; see Table S1, Supplementary Materials) and 3D QSAR modeling performed in Open3DQSAR. Here, models for each of the three bioactivities were computed for a plethora of compound subsets, e.g., separately for the alkaloids of each plant and for various structural subtypes. However, following the above-mentioned working hypothesis, the main goal was to obtain general QSAR models for all or most of the compounds in the dataset.

#### 2.4.1. 3D QSAR Modeling for Cytotoxicity Against L6 Cells

Since an SAR for cytotoxic activity, although distinctly lower than the desired antiprotozoal effects, would be important to understand selectivity, 3D QSAR modeling for this bioactivity was performed first. A 3D QSAR model was initially generated for the cytotoxicity against L6 cells of the complete set of 100 alkaloids. The resulting model was of poor internal predictivity (leave-one-out cross validation (LOOCV) q^2^ = 0.35, i.e., below the acceptable value of 0.5 [17]; see Table 2). This was somewhat improved when only compounds with a normal sterane core were considered, i.e., the 13 compounds with an *abeo*-sterane skeleton were excluded and only 87 regular steranes included. The LOOCV yielded a q^2^ = 0.5, which would be acceptable but not very good. A significant further improvement was noted when compounds of the conanine type (additional ring closure between *N*_20_ and C-18) were also removed from the dataset so that only steranes with a noncyclic side chain at C-17 were considered. The cross-validated coefficient of determination then rose to q^2^ = 0.55 (*n* = 67 compounds). It thus appears that the *abeo*-steranes and the pentacyclic conanines may have (at least partly) different cytotoxicity SARs so that different or additional targets in the cells for these types of compounds may lead to more complex SARs obscuring the linear correlative models when all structural subtypes are combined.

To assess also the potential external prediction power of the 3D QSAR model comprising only the set of 67 regular non-*abeo* and non-conanine steranes, these compounds were then split into various training sets and test sets using different splitting schemes (described under Materials and Methods in Section 3.5) and set sizes. The modeling was repeated independently for each training set and activity predictions subsequently made for the respective test sets. Several of the models obtained yielded q^2^ values and p^2^ values for the test set predictions close to or higher than the acceptable threshold value (0.5), so that their interpretation to extract SAR information appears reasonable. Besides this, validation of the model robustness using the progressive scrambling method [20] implemented in Open3DQSAR yielded normalized fitted Q_0_^2^* values similar to the LOOCV q^2^ values.

Furthermore, to reduce the likeliness that the obtained models were due to chance correlation, the biological data was randomly reassigned to the compounds in three different ways and the model generation repeated (“scramble test”) with the full set of 100 compounds and with the reduced set of 67 regular steranes. In each of these sets, the biological data was randomly assigned in three different ways. None of these six scrambled datasets led to any reasonable model but the PLS modeling failed (no results at all or q^2^ negative or close to 0). Thus, it can be safely assumed that the models obtained with the true bioactivity data do reflect relevant SAR in a realistic manner.

**Figure 4:**
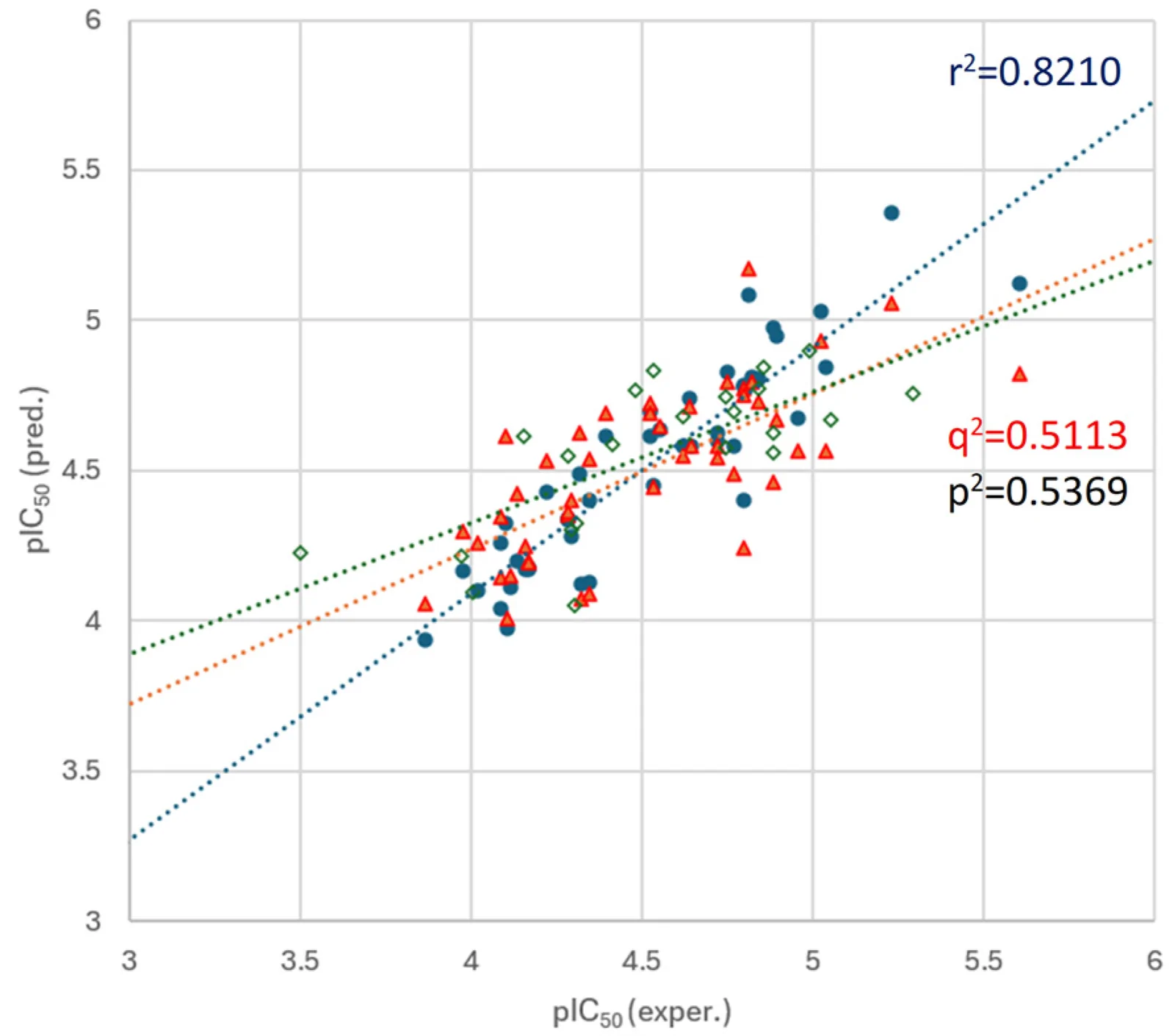
Plot of predicted vs. experimental pIC_50_ values (L6 cytotoxicity) of model L6_67_t2. Training set: PLS calibration: blue dots; LOO CV predictions: red triangles; test set predictions: black cross.

**Figure 5:**
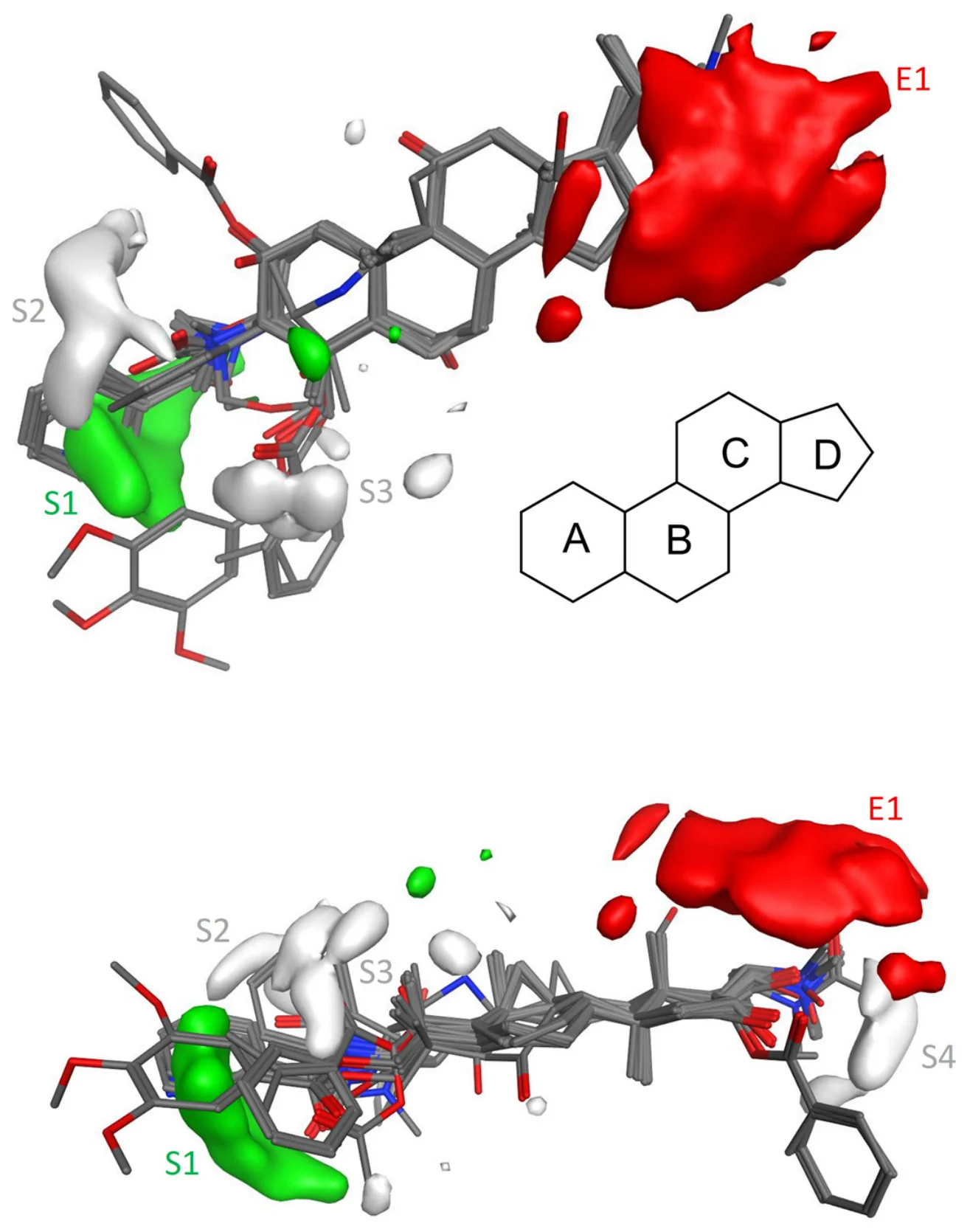
CoMFA maps of model L6_67_t2. All compounds of the training and test set are displayed viewed from two different viewing angles rotated by about 90° relative to each other (compare Figure S2 (Supplementary Materials). Negative and positive contributions of the steric interaction field to cytotoxic activity are mapped as white and green isocontours, respectively. Enhancing effects of electronegative and electropositive substituents on activity are mapped as red and blue isocontours, respectively. (No significant electropositive contributions were found in this model, so that no blue isocontour is visible).

A plot of predicted vs. experimental pIC_50_ values of the best-performing model, L6_67_t2, is shown in Figure 4. The CoMFA maps of model L6_67_t2 are shown in Figure 5.

As obvious in Figure 5, activity-enhancing (green, S1) as well as detrimental (white S2, S3) steric interactions are of major impact in the “west” and “south” regions around ring A (vicinity of C-3 and C-4 substituents, respectively). The enhancing area S1 is more directed towards the a- and the detrimental S2 and S3 regions to the b-side. S1 and S2 are related to substituents on the C-3 amino groups pointing in either direction (methylamino, acetamide, benzamide groups, etc.) which appears to reflect the tendency that a-orientation enhances and b-orientation decreases cytotoxicity. S3 roughly corresponds to the methyl groups at C-4 of many of the *Buxus* AS that show low cytotoxicity. A detrimental steric effect on activity also appears close to ring D, concentrated in the vicinity of C-16 (S4) indicating that substitution at this position will decrease cytotoxicity. A pronounced electrostatic effect (red, E1) dominates the region on the b-side of ring D and the side chain attached to C-17. This area corresponds to a cytotoxicity-enhancing effect of electron-rich substituents in this vicinity such as carbonyl oxygens but also amino groups at position 20 in the side chain.

#### 2.4.2. 3D QSAR Modeling for Antitrypanosomal Activity Against Tbr

Quite notably, a similar observation as in case of the cytotoxicity data was also made when 3D-QSAR models for the anti-*Tbr* activity were generated. Again, removal of *abeo*-pregnanes as well as conanine-type compounds (n = 67) led to a significant improvement of q^2^ from 0.16 (n = 100) to 0.43 (n = 67), see Table 3. However, it was noticed that four compounds (**2-6**, **2-24**, **3-7** and **3-10**) in this reduced dataset obviously still represented outliers due to poor individual cross validation predictions (with absolute residuals >1). After their removal, q^2^ rose to 0.59, so that this dataset of n = 63 compounds was analyzed further. The set was then split into training sets and test sets comprising 33% and 25% of the compounds, using the same splitting schemes as above. Most of the resulting models yielded q^2^ and test set prediction values p^2^ close to or above the acceptable threshold (0.5) so that they can be expected to contain meaningful structure–activity relationship information. Once more, a series of three independent random scramble tests was performed with the total set of 100 compounds as well as the reduced set of 63 aminosteroids, which only resulted in failure of model generation so that chance correlation was unlikely. Furthermore, progressive scrambling as implemented in open3Dqsar was also performed, yielding normalized fitted Q_0_^2^* values similar to the normal LOO q^2^ values as would be expected for robust models [18,20].

**Figure 6:**
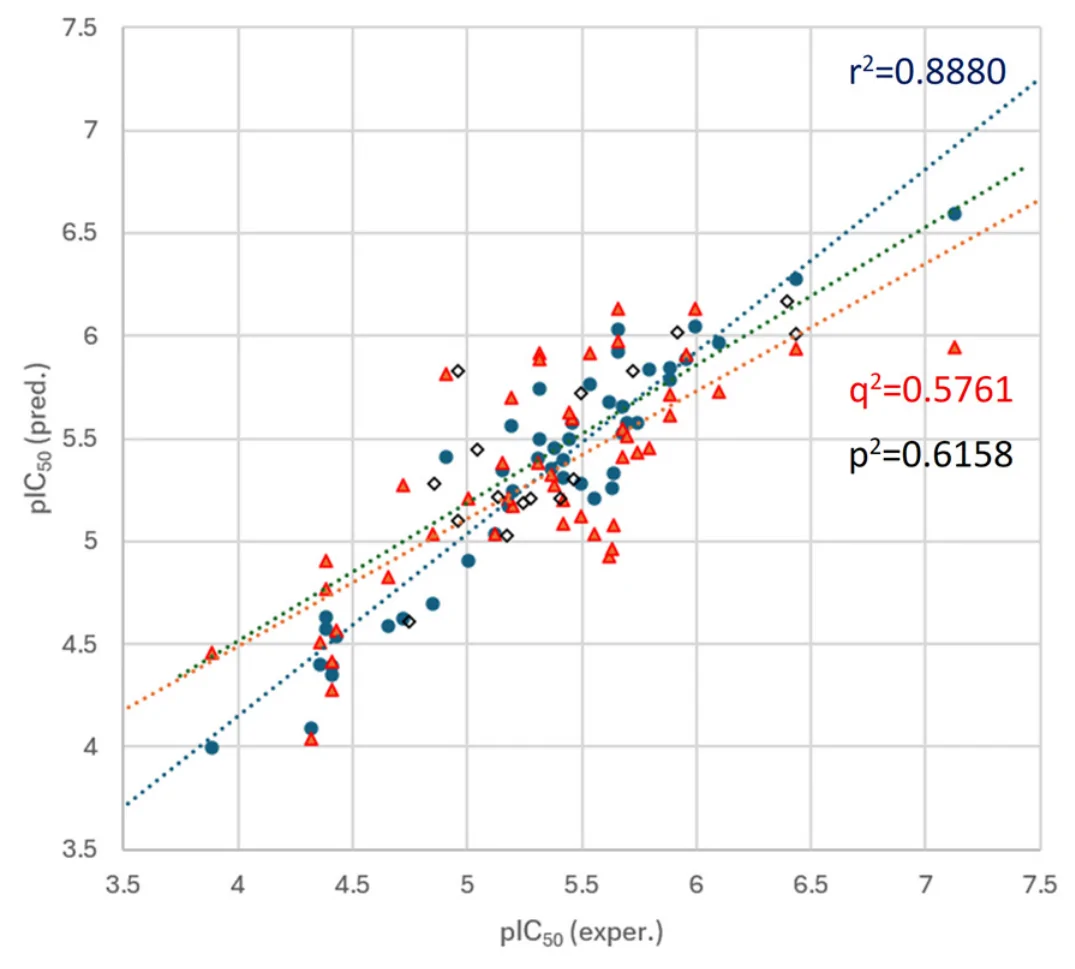
Plot of predicted vs. experimental pIC_50_ values (anti-*Tbr* activity) of model *Tbr*_63_t6. Training set: PLS calibration: blue dots; LOO CV predictions: red triangles; test set predictions: black diamonds.

A plot of predicted vs. experimental pIC_50_ values of the best-performing model, *Tbr*_63_t6, is shown in Figure 6. As both, the internal cross validation as well as the external test set predictions, yield quite satisfactory fit with coefficients of determination in the range of 0.6, this model appeared suitable for detailed interpretation.

**Figure 7:**
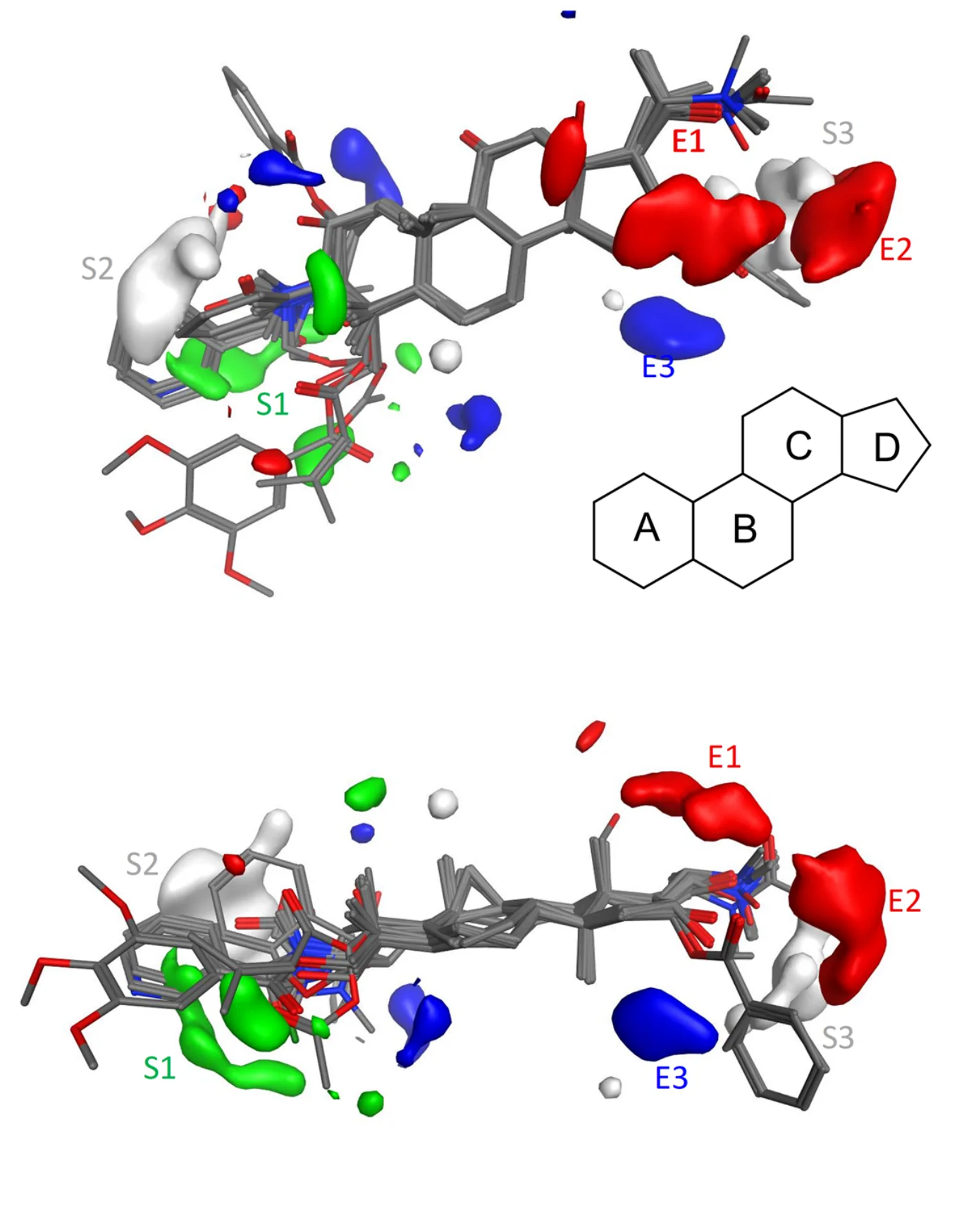
CoMFA maps of model *Tbr*_63_t6. All compounds of the training and test set are displayed viewed from two different viewing angles rotated by about 90° relative to each other (compare Figure S2 (Supplementary Materials). Negative and positive contributions of the steric interaction field to antitrypanosomal activity are mapped as white and green isocontours, respectively. Enhancing effects of electronegative and electropositive substituents on activity are mapped as red and blue isocontours, respectively.

The underlying CoMFA maps with the major contributions of the steric and electrostatic MIFs to this model are depicted in Figure 7. Steric interactions with negative impact on anti-*Tbr* activity occur on both the A- and D-ring ends (bulky substituents in the C-3 (b) and C-16 vicinity: S2 and S3, respectively) whereas an activity-enhancing steric interaction (S1) appears close to the former region but on the a side. Electrostatic effects appear to be concentrated around the D-ring with several red patches (E1, E2) where electron-rich substituents enhance activity on the b-face and a smaller one (E3) on the a-face with an opposite impact of electronegativity on activity. A few small electropositive (blue) areas of unclear significance also appear around the A and B rings. See Section 2.4.4 for comparison with the models for cytotoxicity and antiplasmodial activity.

#### 2.4.3. 3D QSAR Models for Cytotoxic and Antitrypanosomal Activity of Conanine-Type Alkaloids

It was striking to note that removal of the compounds with non-regular sterane carbocyclic core structures (*abeo*-sterane and conanine-type skeletal types) led to very significant improvement of the 3D-QSAR models for the anti-*Tbr* and cytotoxic activity of the remaining regular steranes (including nor-cycloartanes). On the other hand, the question arose whether these compounds might share different SARs amongst their groups which might point towards (at least partly) different molecular targets. Since only 13 *abeo*-steranes were included in the dataset representing only a rather narrow span of activity (<2 log units for the anti-*Tbr* and <1 for the L6 cytotoxic activity) at an overall mediocre activity level, it did not appear feasible to construct separate QSAR models for them. In the case of the 20 conanine-type *Holarrhena*-alkaloids, some of whom displayed antiprotozoal activity and selectivity at a very promising level, the span of anti-*Tbr* pIC_50_ values was reasonably large (covering more than 2.5 log units), so that it appeared statistically feasible to attempt the construction of a 3D-QSAR model.

**Figure 8:**
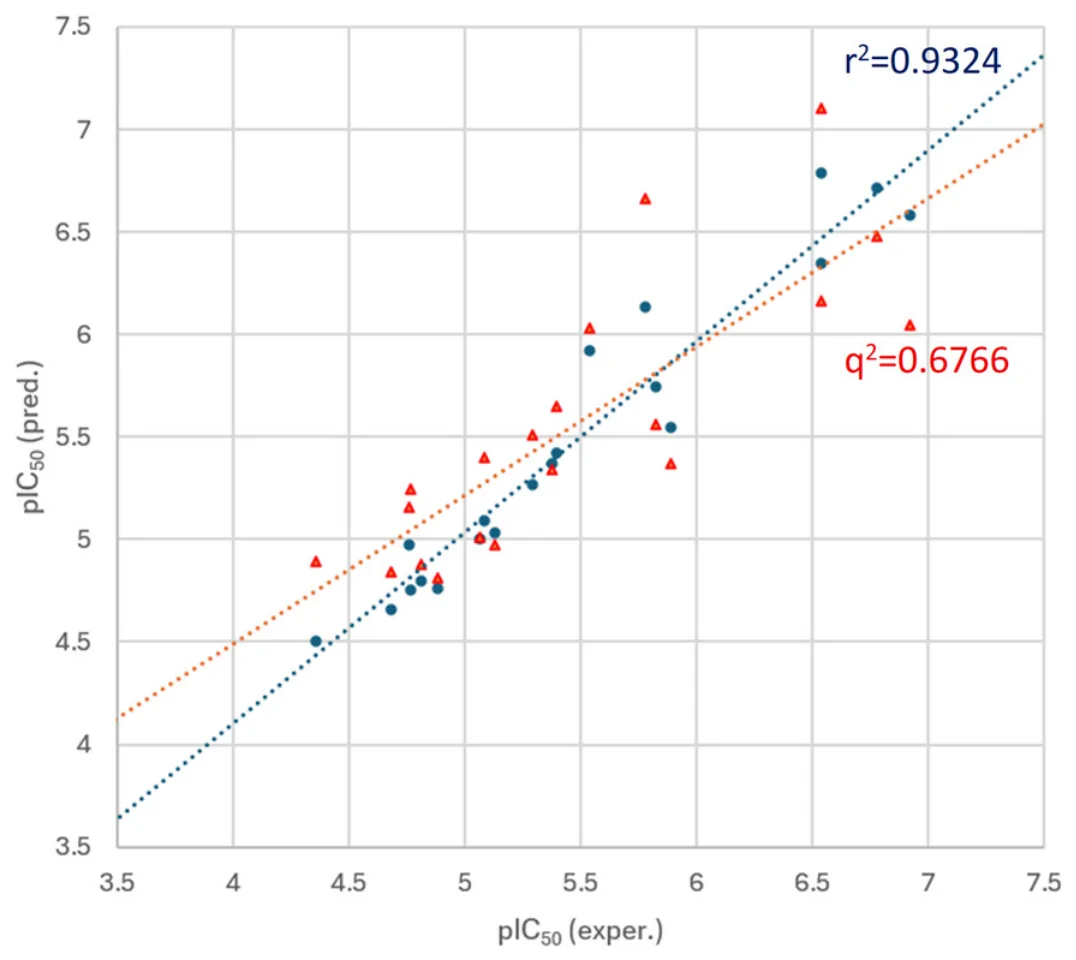
Plot of predicted vs. experimental pIC_50_ values (anti-*Tbr* activity) of the 20 conanine-type AS (model *Tbr*_20_con). Training set: PLS calibration: blue dots; LOO CV predictions: red triangles.

**Figure 9:**
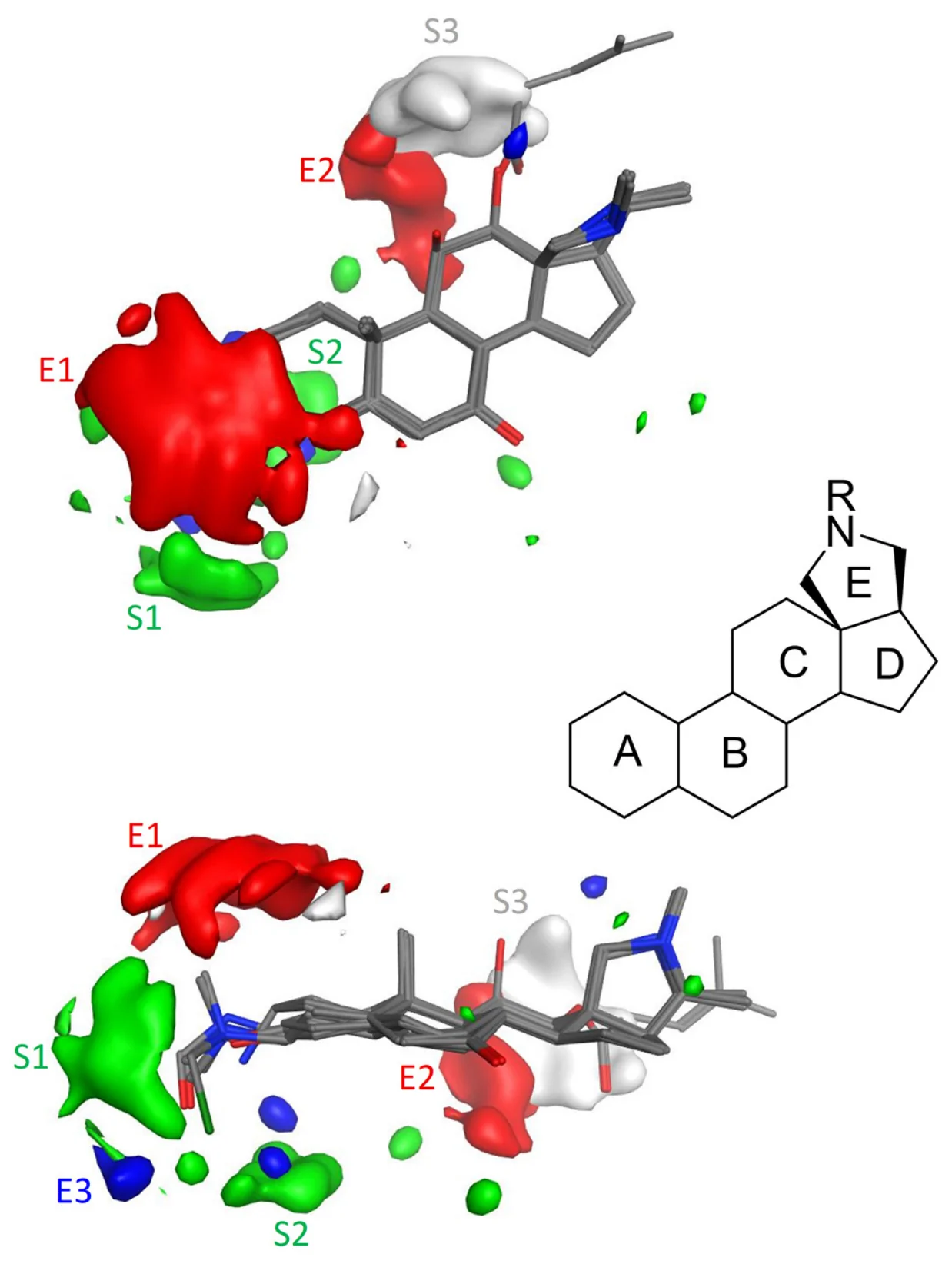
CoMFA maps of model *Tbr*_20_con. All compounds are displayed viewed from two different viewing angles rotated by about 90° relative to each other (compare Figure S2 (Supplementary Materials)). Negative and positive contributions of the steric interaction field to antitrypanosomal activity are mapped as white and green isocontours, respectively. Enhancing effects of electronegative and electropositive substituents on activity are mapped as red and blue isocontours, respectively.

Indeed, the entire set of 20 compounds in this case yielded a model with a satisfactory LOO CV q^2^ value of 0.68 and also a reasonable Q_0_^2^* value of 0.57 from progressive scrambling (see Table 4). This set of compounds was therefore also split into training and test sets with the training set comprising either 2/3 or 3/4 of the compounds (i.e., 13 or 15 compounds, with 7 or 5 test set compounds, respectively), using the same three different splitting schemes as above. However, the models obtained in these calculations, probably due to the small number of compounds, then suffered from either unacceptably poor results of the LOO CV and progressive scrambling tests or too poor external predictive potency. However, since the overall model obtained with all 20 compounds as training set gave at least acceptable internal predictions (see Figure 8), an interpretation of the underlying molecular interaction maps should be allowed with due caution (see Figure 9).

An attempt to construct a 3D QSAR model also for the cytotoxic activity of these 20 conanines led to a model with a mediocre q^2^ value of only 0.42 and an even lower Q_0_^2^* (Table 4). Further optimization of this model was not attempted due to the small number of compounds in the set.

In comparison with the model for anti-*Tbr* activity of the AS with a sterane/pregnane skeleton without the additional conanine heterocycle (compare Figure 7), the interaction profile of the conanines shown in Figure 9 appears conspicuously different. An extensive area of favorable electronegative interactions (E1, red) is observed above the “southwestern” parts of ring A, accompanied by a second region with the same property near C-11/C-12 in ring C (E2, see below). Neither of these are present in the pregnane model. This model’s most prominent electronegative interaction around ring D, instead, is missing here. The steric preferences also appear to differ significantly between the two types of AS: While the favorable (green) region of interaction around the C-3 position in ring A (S1, S2) is more pronounced than in case of the pregnanes, the conanines display a distinct area of unfavorable steric interaction S3 in the vicinity of C-12, directly neighboring E2 mentioned above. These latter contours are caused by the C-12 OH and O-acyl substituents in compounds **4-14**–**4-17**. Compounds **4-14** and **4-15** bear a hydroxy group which is esterified in **4-16** and **4-17**. The alcohols being less active than the esters, the red region E2 appears to reflect an enhancing impact of the ester oxygens on activity and a deleterious effect of the electropositive OH-proton at the same time. These substituents occur only in the conanine series (compounds), so that their significance in distinguishing the two subsets cannot be assessed.

#### 2.4.4. 3D QSAR Modeling for Antiplasmodial Activity Against Pf

In case of the malaria parasite *Pf*, biological data were only available for 87 compounds since this activity was not determined for the compounds from our study on *Holarrhena floribunda* (*H. africana*) [10]. 3D QSAR modeling with the whole dataset in this case did not yield a meaningful model (n = 87, r^2^ = 0.64, q^2^ < 0.1, Table 5). It turned out that confinement of the dataset to only regular steranes (i.e., removal of *abeo*-steranes and conanine-type compounds as in case of cytotoxic and anti-*Tbr* activity) did not yield an improvement of the statistical quality. However, it was noticed that two compounds, the chlorinated derivatives **3-5** and **5-8**, represented strong outliers in the total dataset of 87 compounds. Thus, when reduced to 85 by removal of the two chloromethylamino derivatives, the q^2^ value was improved to 0.41. Removal of three further outliers with absolute residuals of their LOOCV predictions >1 (**1-8**, **2-4** and **3-17**) then led to improvement of q^2^ with the reduced dataset of n= 82 compounds well into the accepted range (q^2^ = 0.58). This set was then further validated by test set predictions for 1/3 and 1/4 of the compounds as before, yielding various models with satisfactory internal and external predictive performance. Furthermore, progressive scrambling as implemented in Open3DQSAR was also performed, yielding normalized fitted Q_0_^2^* values very similar to the normal LOO q^2^ values as would be expected for robust models (see Table 5).

**Figure 10:**
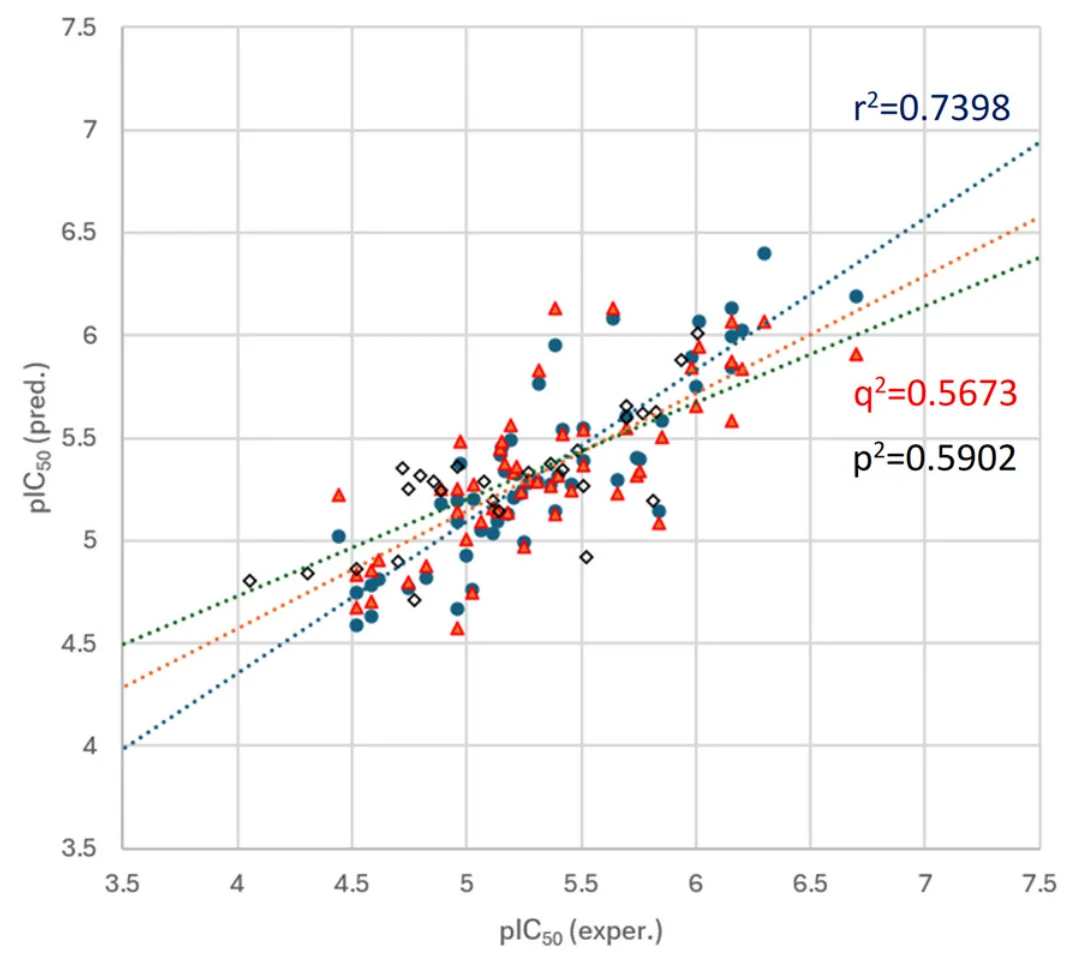
Plot of predicted vs. experimental pIC_50_ values (anti-*Pf* activity) of model *Pf*_82_t2. Training set: PLS calibration: blue dots; LOO CV predictions: red triangles; test set predictions: black diamonds.

**Figure 11:**
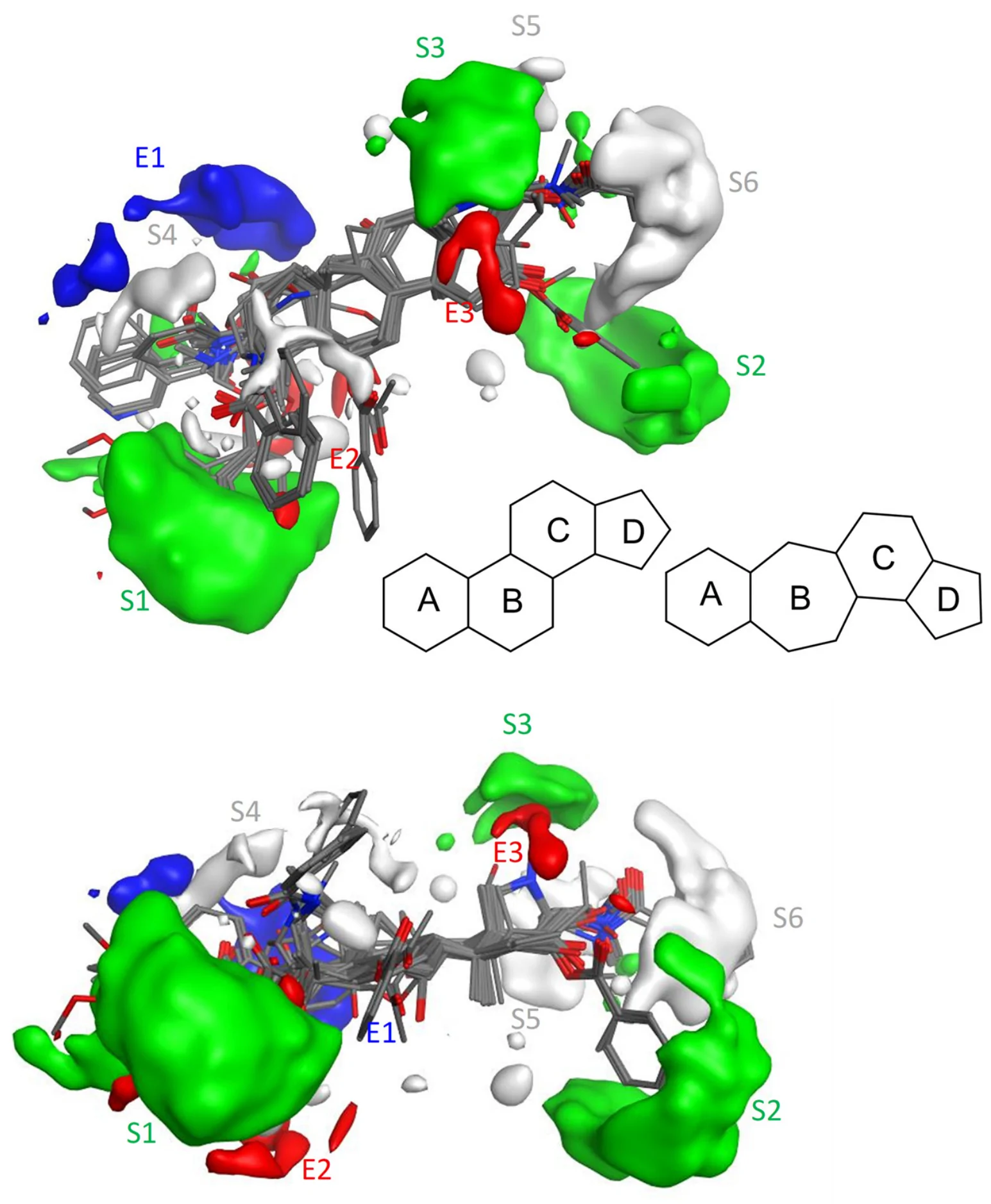
CoMFA maps of model *Pf*_82_t2. All compounds of the training and test set are displayed viewed from two different viewing angles rotated by about 90° relative to each other (compare Figure S2 (Supplementary Materials)). Negative and positive contributions of the steric interaction field to antiplasmodial activity are mapped as white and green isocontours, respectively. Enhancing effects of electronegative and electropositive substituents on activity are mapped as red and blue isocontours, respectively.

A plot of predicted vs. experimental pIC_50_ values of the best-performing model, *Pf*_82_t2, is shown in Figure 10. It is obvious that the predictions with this model are far from optimal but nevertheless quite reasonable, given that the model consists of only two PLS components. It was therefore analyzed with respect to the underlying MIF contributions. The CoMFA maps of model *Pf*_82_t2 are shown in Figure 11.

This model displays a very conspicuous activity-enhancing steric region (green, S1) around the area of substituents in the C-4 neighborhood (A ring, “south side”), where bulky acyl substituents are found in several of the compounds with high activity against *Pf*, such as compounds **2-5** or **3-6**. Another region with this effect is observed in the vicinity of C-16 substituents on the “east” and a-side of ring D (S2). This is where the tigloyl side chains as well as benzoyl ester moieties of some of the more active *Buxus*-alkaloids such as **1-6** or **1-2** extend. A third significant green patch is on the beta side of ring D, where the *N*-methyl groups of various conanines with strong activity (e.g., **5-7**, **5-5**, **5-9**) extend. Regions of negative steric interaction are observed in the region of the C-17 side chain (S5, S6) of which especially the latter interacts with bulky side chain substituents of the least active compounds such as **3-14**. In the electrostatic field, a relatively prominent blue region (where electropositive substitution would enhance activity, i.e., electron-rich substituents would show a detrimental effect) is located in the vicinity of C-2 in ring A (E1). This appears to be due to the electron-rich OH groups at C-2 in several of the less active compounds from *B. obtusifolia*. A smaller red patch on the a-side of ring A (E2) is in a region where some of the more active compounds extend electron-rich groups such as 3a-amino groups (e.g., **3-6**, **3-10**, **5-7**, etc.) or amide carbonyl oxygens (e.g., **3-3**, **5-9**). In the neighborhood of ring D, a small red region on the beta side (E3) corresponds to the activity-enhancing effect of the electron-rich cyclic amino group of conanine-type AS, some of which (**5-9**, **5-7**, **5-5**) are among the most active antiplasmodial compounds in the series. At the same time, the nearby green steric patch S3 corresponds to these amino groups’ methyl substituent which is hence also predicted to enhance anti-*Pf* activity.

#### 2.4.5. Comparison of 3D QSAR Models for Cytotoxic, Antitrypanosomal and Antiplasmodial Activity

Although it is likely that apparent differences between the various 3D-QSAR models may be influenced by the fact that different sets of compounds had to be used to obtain them, a careful comparison should be allowed under this caveat.

**Figure 12:**
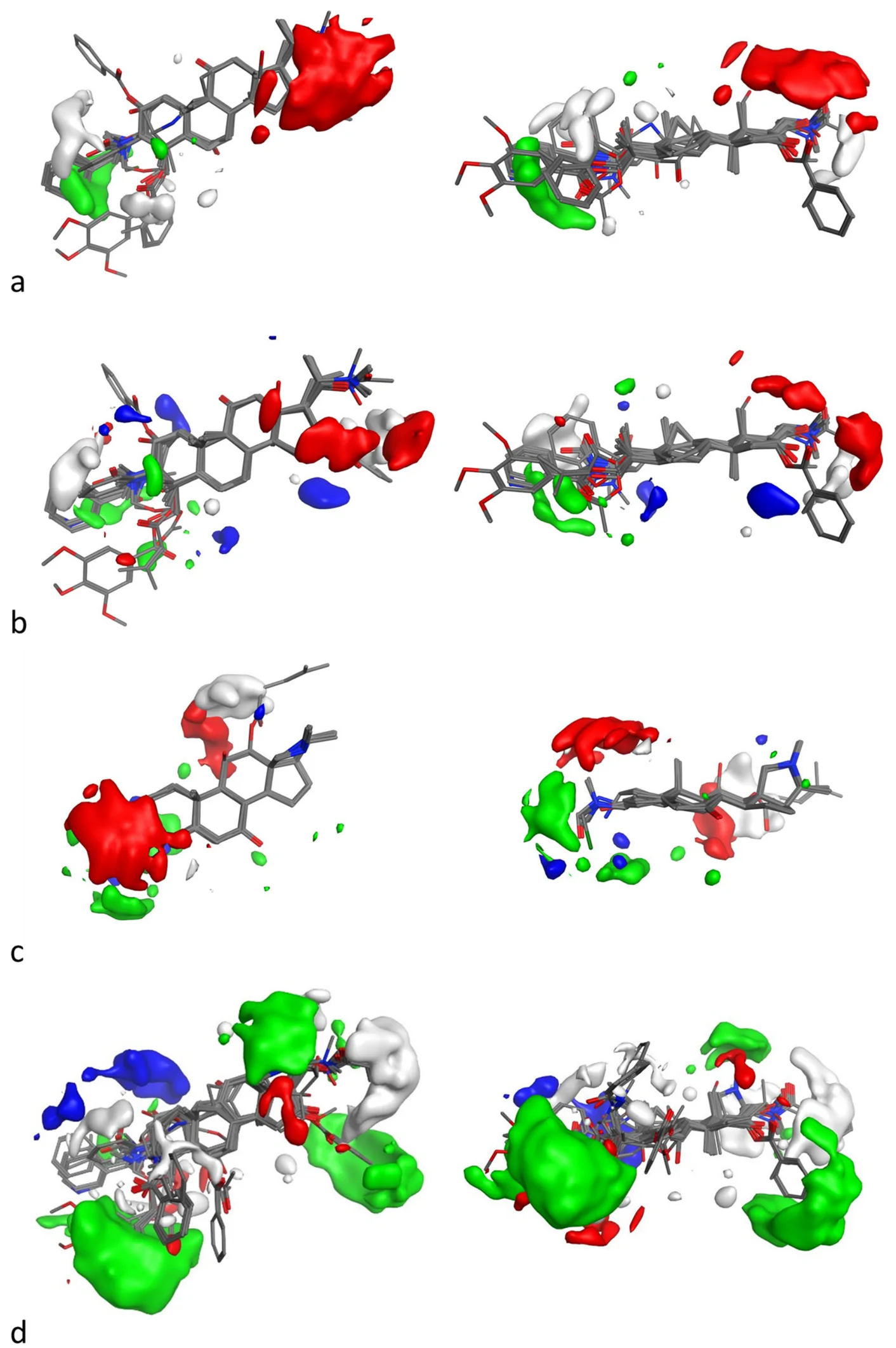
Comparison of 3D QSAR models for L6 cytotoxicity (**a**), anti-*Tbr* activity (**b**,**c**): models for regular pregnanes and conanines, respectively) and anti-*Pf* activity (**d**). The CoMFA maps are shown separately and from two different viewing angles (left and right, compare Figure S2, Supplementary Materials) in each case. Green and white isocontours represent enhancing and detrimental effects of steric interactions on activity while red and blue isocontours represent activity-enhancing electrostatic effects of electronegative/electron-rich and electropositive/electron deficient substituents, respectively.

To identify potential interaction regions responsible for the observed differences in activity and thus to obtain explanations for selectivity, the CoMFA maps of the different models are shown in direct comparison in Figure 12.

It is quite plain to notice a relatively high degree of similarity between the model for cytotoxicity and for anti-*Tbr* activity of the AS with regular pregnane skeleton (Figure 12a and Figure 12b, respectively). The positive (green) steric interactions around ring A as well as the negative effects (white) around this ring and the C-16 vicinity in ring D appear very similar. Furthermore, a pronounced electronegative (red) area above the D-ring is common to both models showing a related electrostatic interaction profile as well.

The interaction profile antitrypanosomal model of the conanine-type AS (Figure 12c) shows a certain commonality with the aforementioned CoMFA maps in the favorable (green) steric region in the C-3 vicinity around the “southwest” of ring A. However, it differs fundamentally from the other models as it displays a strong electronegative (red) effect right above ring A while lacking the one above ring D. Instead, it features another, smaller, region of this type near C-11, accompanied by a negative steric (white) interaction close by, just “north” of ring C. As already stated above, these two last mentioned features are related to the compounds with a C-12-OH or O-acyl moiety that does not occur in the other series of AS, so that its relevance in distinguishing between the series’ interaction patterns is not clear.

Whatsoever, the existence of two clearly distinct SARs for AS with an acyclic pregnane-type sidechain at C-17 on one hand and the conanines with their additional pyrrolidine ring on the other indicates that there is a difference between the targets or, at least, in the binding mode, if the target(s) is/are the same. It is important to recall that a model containing both types of AS performed much worse than the separate models which is a good indicator of the existence of distinct SARs and MoAs. On the other hand, we have previously shown that it is possible to construct meaningful 3D QSAR models for the anti-*Tbr* activity and cytotoxicity of the subset of AS from *Holarrhena africana* [13], which consists of conanines (n = 11) and normal pregnanes (n = 6) with a simple C_2_-sidechain at C-17, namely an acetyl group. The latter, apparently, did not cause a problem in constructing a common QSAR with the conanines. This may be due to the fact that they all have the same acetyl moiety at C-17 while the new extended set of AS contains a much wider chemical diversity in this sidechain. It is straightforward to assume that the observed difference in selectivity, *Holarrhena* conanines being far more selective than their C-17-acetyl-sidechain congeners, is a consequence of the postulated difference in molecular targets or binding mode. In any case, the conanine-type AS represent the most promising subset in this series for potential further development into an anti-*Tbr* drug.

The 3D-QSAR model for antiplasmodial activity (Figure 12d), on the other hand, appears fundamentally different from all others at first sight, being strongly dominated by very pronounced steric effects. Three very large favorable (green) regions of steric interaction, in the “southwest” of ring A as well as above the additional conanine ring E and around the a-side of ring D are accompanied by a strong negative steric area near the latter, but more to the b-side of ring D, directed towards the pregnane-AS sidechain. The favorable steric pockets are clearly due to several of the most potent AS extending bulky aromatic or aliphatic acyl moieties into these regions. The electronegative (red) patch observed in the cytotoxic and anti-*Tbr* models (Figure 12a,b) in this model has shrunken to a rather small spot just above the D-ring and a relatively strong blue (electropositive) region appears instead on the “north” side of the A-ring, in the vicinity of C-1 and C-2.

This fundamentally different interaction pattern indicates that the antiplasmodial activity of the AS under study follows a decisively different SAR and is hence more likely to be caused by attack on a different target from those responsible for the antitrypanosomal and cytotoxic effects. This is also supported by the fact that representatives of all the subsets under study (i.e., regular steranes with an acyclic sidechain at C-17, conanines as well as *abeo*-sterane dervivatives) could be combined in a statistically satisfactory model, which would not be unlikely if the underlying SAR were more closely related to those guiding the anti-*Tbr* and cytotoxic activities. The series, with its most active antiplasmodial congeners such as **1-6**, **1-7**, **2-5** and **3-6**, thus represents a very promising starting point for new selective antimalarial leads. Here, it appears very promising to investigate congeners possessing combinations of aromatic or olefinic groups extending into more than one of the pockets of favorable steric interaction.

## 3. Materials and Methods

### 3.1. Origin and Isolation of the Investigated Compounds, Biological Testing and Bioactivity Data

All compounds were isolated in our group. Their isolation from *Buxus sempervirens* (**1-1**–**1-24**) [6,7], *Buxus obtusifolia* (**2-1**–**2-24**) [8], *Pachysandra terminalis* (**3-1**–**3-18**) [9], *Holarrhena africana* (=*H. floribunda*; **4-1**–**4-17**) [10] and *Holarrhena pubescens* (**5-1**–**5-20**) [11], as well as identification and structure determination has been described in detail previously.

### 3.2. Biological Testing and Bioactivity Data

The activity testing of all compounds has been reported previously in the original publications [6,7,8,9,10,11]. The original bioactivity data, IC_50_ concentrations in µmol/L, as common in QSAR studies, were converted to negative decadic logarithms of these concentrations expressed in mol/L (–log (IC_50_ [mol/L]) = pIC_50_ values). Thereby, the potency within each dataset can be directly compared on a linear scale with the highest activity represented by the highest pIC_50_ value. In cases where the same compound had been isolated twice and tested independently, the arithmetic mean of the pIC_50_ values was used. All pIC_50_ values are reported in Table S1, Supplementary Materials.

### 3.3. ADME Calculations

The ADME properties of all compounds were obtained online using the SwissADME online tool [14]. To this end, a list of all SMILES representations was uploaded and the resulting output downloaded from the web tool.

### 3.4. Molecular Modeling

Molecular models were generated with the Molecular Operations Environment (MOE, v. 06.2024; Chemical Computing Group, Montreal, QC, Canada [19]). Each compound’s structure was built in MOE in the absolute configuration shown in Figure 1 and energy minimized using the MMFF94X force field. Amino groups were modeled in their neutral, i.e., unprotonated state. This does not imply that this is the bioactive form. However, all respective compounds were treated identically. The ammonium salts could be expected to yield roughly opposite electrostatic interaction maps than the free bases. A conformational search was performed with the low-mode molecular dynamics (LMD) method with standard default settings. The resulting lowest-energy conformers of each compound were first manually superposed using a 5-point alignment of the steroid cores as previously described [13]. This alignment was then refined using the flexible alignment method (flexalign.svl) implemented in MOE. Default settings were used, except that the maximum number of atoms in the calculation had to be increased by factor 4 (from 4095 to 16380) in the underlying program module flexalgn.svl in order to allow simultaneous treatment of all 100 structures. Atomic partial charges were computed for each model using the semi-empirical AM1 method as implemented in MOE/MOPAC. All 3D models thus obtained in their optimal alignment were finally stored in a joint MOE database where they were combined with their biological data for further QSAR calculations. No realignment was performed with the reduced datasets.

### 3.5. 3D QSAR Modeling

3D QSAR models were generated with the open3Dqsar software, v. 2.281 [18]. The standard settings for grid spacing and molecular field calculations (steric and electrostatic) were applied. The overall procedure code is reported in the Supplementary Materials (Program execution code S1).

The calculated bioactivity values for each of the PLS models (PLS calibration and leave-one-out cross validation (LOOCV) as well as test set prediction (PRED)) were saved in MOE databases for subsequent analysis. Likewise, each model’s PLS coefficient maps for the steric and electrostatic interaction fields were exported to MOE grid files for visualization and analysis in MOE.

All models were validated by leave-one-out cross validation (LOOCV) and the internal predictive quality assessed by the resulting q^2^ values (coefficient of determination of internal predictions after LOOCV). The optimal number of significant latent variables (LV, PLS components) for each model was chosen so that if addition of a further LV did not further increase q^2^, no further LVs were added. In cases of datasets with reasonable q^2^ values (near 0.5 or greater), external predictions were made for test sets comprising one third and one quarter of the dataset and the prediction assessed on grounds of the coefficient of determination of the predicted vs. experimental data. The distribution of compounds into the training sets and test sets was based on three different schemes: In each case, the dataset under investigation was sorted in decscending order by the bioactivity (pIC_50_ values) under study and then three molecular fingerprints (1: typed atom triangles, 2: 3-point pharmacophore and 3: 4-point pharmacophore) were used to compute diverse subsets as implemented in MOE. The dataset was then sorted by these three diverse subsets individually so that, in each case, the 2/3 or 3/4 most diverse structures could be assigned the training sets leaving the remaining 1/3 or 1/4 as the test sets t1-t3 and t4-t6, respectively. Models 1–6 were then computed with the training sets (including LOOCV) and external predictions for the respective test sets made after completion of the models.

Additionally, all models chosen for external validation were also subjected to further validation by progressive scrambling as implemented in Open3DQSAR [20].

Furthermore, representative datasets were also subjected to a set of three random scrambling tests [21] to reduce the likeliness of chance correlations. To this end, the respective bioactivity data were randomly reassigned the structures in three different ways in each case and the model calculations attempted with these false data. These tests proved successful in all cases, i.e., no models resulted from these computations or the results yielded irrelevant q^2^ values.

## 4. Conclusions

The present study presents a comprehensive investigation of 3D quantitative structure–activity relationships for the antitrypanosomal, antiplasmodial and cytotoxic activities of a set of 100 chemically related aminosteroids obtained from five plant species of the families Apocynaceae and Buxaceae. 3D-QSAR modeling revealed that the activity against *Trypanosoma brucei rhodesiense* for most of the compounds follows similar structural requirements as the cytotoxicity against mammalian (L6) cells. This is fundamentally different for the subset of conanine-type aminosteroids from *Holarrhena* species possessing an additional pyrrolidine ring. A very divergent interaction pattern was found to explain these compounds’ anti-*Tbr* activity in comparison with that of the bulk of the other AS. This discrepancy can be considered the basis for the conanines’ observed high level of selectivity against this parasite and renders them excellent candidates for further development of potential antitrypanosomal leads or drugs.

The interaction pattern observed in the 3D-QSAR model for antiplasmodial activity of the majority of AS shows conspicuous differences from the models for antitrypanosomal and cytotoxic activity. This activity appears to be governed much more prominently by the molecules’ ability to engage in “steric” (i.e., van der Waals, hydrophobic or p-p) interactions with target(s) probably very different from those involved in the other bioactivities. Bulky aromatic moieties in various positions around the steroid scaffold appear to enhance the antiplasmodial activity, which could be a good starting point for further structural improvement towards even more active and selective antiplasmodials. In summary, the present investigation paves the way towards rational optimizations of this promising class of antiprozozoal agents.

## Figures and Tables

**Table 1:** Overview of the distribution of pharmacologically relevant physicochemical and ADME properties in the set of 100 aminosteroids under study as obtained from the Swiss-ADME online tool [14] along with the distribution of bioactivity data (−log IC_50_ (mol/L) = pIC_50_ values).

	pIC_50_											CYP Inhibitor **								
	*Tbr*	*Pf*	L6	MW	HBA ^a^	HBD ^b^	Cons. log P ^c^	Cons. sol. ^d^	GI abs. **	BBB per. **	Pgp subs **	1A2	2C19	2C9	2D6	3A4	n Lip viol.	Lip * fail.	Bioav. score	PAINS alerts
				500 *	10 *	5 *	5 *													
max	7.125	6.745	5.606	654.8	8	3	6.160	2	1	1	1	1	1	1	1	1	2	1	0.550	0
median	5.313	5.252	4.475	399.6	3.000	1.000	4.260	1.000	1.000	1.000	0.000	0.000	0.000	0.000	0.000	0.000	1.000	0.000	0.550	0.000
mean	5.342	5.304	4.461	413.3	3.130	1.420	4.412	0.827	0.960	0.770	0.470	0.030	0.010	0.030	0.080	0.060	0.819	0.100	0.512	0.000
min	3.888	4.053	3.500	312.5	1	0	2.800	0	0	0	0	0	0	0	0	0	0	0	0.170	0
								counts (*n* compounds)												
								non-solub.	non-abs.	non-per.	Pgp subs.	1A2 inh.	2C19 inh.	2C9 inh.	2D6 inh.	3A4 inh.	Lip. viol. > 0	Lip. fail.	Bioav. score ≥0.55	*n* PAINS
								29	4	23	47	3	1	3	8	6	71	10	90	0

^a^ number of hydrogen bond acceptors; ^b^ number of hydrogen bond donors; ^c^ Consensus log P = mean of logP values calculated by 5 different algorithms; ^d^ Consensus solubility = derived from solublity estimates (2 = soluble, 1 = moderately soluble, 0 = insoluble) calculated by 3 different algorithms; * Maximum threshold values of Lipinski rule of 5. ** Binary representation: 1 = yes, 0 = no; GI abs.: Gastrointestinal absorbable; BBB per.: Blood–brain barrier permeable; Pgp subs. P-glycoprotein substrate; CYP inhibition of Cytochrome -P450 isoenzymes; nLip viol: Number of violations of Lipinski rule of five; Lip fail: Failure of Lipinski rule of five (i.e., ≥2 violations); Bioav. Score: SwissADME bioavailabllitiy score; PAINS alerts: Alerts for “pan assay interference compounds”.

**Table 2:** Statistical characteristics of 3D QSAR models for cytotoxic activity against L6 myoblasts. The model with the best statistical performance, marked by bold characters, was chosen for further analysis.

Model Code	Dataset Definition	Train:Test	n cpds	PLS Comp	r^2^	q^2^	p^2^	Q_0_^2^*
L6_100	all (reg. ster. + abeo)	100:0	100	5	0.70	0.35	-	
L6_87	reg. ster. (no abeo)	87:0	87	5	0.76	0.50	-	
L6_67	reg. ster., no con.	67:0	67	5	0.80	0.55	-	
test set 1/3								
L6_67_t1	reg. ster., no con.	45:22	67	4	0.82	0.41	0.44	0.36
**L6_67_t2**	**reg. ster., no con.**	**45:22**	**67**	**4**	**0.82**	**0.51**	**0.54**	**0.45**
L6_67_t3	reg. ster., no con.	45:22	67	4	0.85	0.39	0.56	0.33
test set 1/4								
L6_67_t4	reg. ster., no con.	50:17	67	3	0.78	0.51	0.11	0.50
L6_67_t5	reg. ster., no con.	50:17	67	4	0.82	0.48	0.46	0.46
L6_67_t6	reg. ster., no con.	50:17	67	2	0.70	0.38	0.40	0.31

**Table 3:** Statistical characteristics of 3D QSAR models for antiprotozoal activity against *Tbr*. The model with the best statistical performance, marked by bold characters, was chosen for further analysis.

Model Code	Dataset Definition	Train:Test	n cpd	PLS cmp	r^2^	q^2^	p^2^	Q_0_^2^*
*Tbr*_100	all (reg. ster. + abeo)	100:0	100	5	0.64	0.16	-	
*Tbr*_87	reg. ster. (no abeo)	87:0	87	5	0.67	0.28	-	
*Tbr*_67	reg. ster., no con.	67:0	67	5	0.80	0.43	-	
*Tbr*_63	reg. ster., no con., no outl.	63:0	63	5	0.84	0.59	-	
test set 1/3								
*Tbr*_63_t1	reg. ster., no con., no outl.	42:21	63	5	0.86	0.47	0.46	0.43
*Tbr*_63_t2	reg. ster., no con., no outl.	42:21	63	5	0.89	0.51	0.42	0.43
*Tbr*_63_t3	reg. ster., no con., no outl.	42:21	63	5	0.89	0.46	0.62	0.50
test set 1/4								
*Tbr*_63_t4	reg. ster., no con., no outl.	47:16	63	5	0.89	0.64	0.42	0.64
*Tbr*_63_t5	reg. ster., no con., no outl.	47:16	63	5	0.85	0.50	0.49	0.46
***Tbr*** **_63_t6**	**reg. ster., no con., no outl.**	**47:16**	**63**	**5**	**0.89**	**0.57**	**0.62**	**0.57**

**Table 4:** Statistical characteristics of 3D QSAR models for activity of 20 conanine-type AS against *Tbr* and their cytotoxicity against L6 cells. Due to poor validation results (at least one parameter unacceptable; red) of the test set validated models, only the anti-*Tbr* model with all 20 compounds as training set (bold characters) was chosen for (cautious) further analysis.

Model Code	Dataset Definition	Train:Test	n cpd	PLS cmp	r^2^	q^2^	p^2^	Q_0_^2^*
***Tbr*_20_con**	**conanine-type AS**	**20:0**	**20**	**5**	**0.93**	**0.68**	**-**	**0.57**
L6_20_con	conanine-type AS	20:0	20	4	0.85	0.42	-	0.25
test set 1/3								
*Tbr*_20_t1	conanine-type AS	13:7	20	5	0.95	0.50	0.75	0.12
*Tbr*_20_t2	conanine-type AS	13:7	20	5	0.97	0.34	0.31	−0.02
*Tbr*_20_t3	conanine-type AS	13:7	20	5	0.99	0.43	0.35	0.09
test set 1/4								
*Tbr*_20_t4	conanine-type AS	15:5	20	5	0.94	0.67	0.14	0.67
*Tbr*_20_t5	conanine-type AS	15:5	20	5	0.95	0.22	0.29	−0.2
*Tbr*_20_t6	conanine-type AS	15:5	20	5	0.94	0.23	0.40	−0.1

**Table 5:** Statistical characteristics of 3D QSAR models for antiprotozoal activity against *Pf*. The model with the best statistical performance, marked by bold characters, was chosen for further analysis.

Model Code	Dataset Definition	Train: Test	n cpd	PLS cmp	r^2^	q^2^	p^2^	Q_0_^2^*
*Pf*_87	all (reg. ster. + abeo)	87:0	87	1	0.64	<0.1	-	
*Pf*_85	all, no chlorinated	85:0	85	3	0.67	0.41	-	
*Pf*_82	all, no chlorin., no outl.	82:0	82	3	0.72	0.58	-	
test set 1/3								
*Pf*_82_t1	all, no chlorin., no outl.	55:27	82	2	0.68	0.45	0.67	0.46
***Pf*** **_82_t2**	**all, no chlorin., no outl.**	**55:27**	**82**	**2**	**0.74**	**0.57**	**0.59**	**0.55**
*Pf*_82_t3	all, no chlorin., no outl.	55:27	82	2	0.73	0.55	0.52	0.52
test set 1/4								
*Pf*_82_t4	all, no chlorin., no outl.	61:21	82	2	0.63	0.39	0.82	0.41
*Pf*_82_t5	all, no chlorin., no outl.	61:21	82	3	0.76	0.56	0.53	0.53
*Pf*_82_t6	all, no chlorin., no outl.	61:21	82	2	0.73	0.57	0.47	0.55

## Data Availability

The original data underlying this study were published previously [6,7,8,9,10,11]. All computational details (numerical output of all computations, including molecular models, 3D QSAR models and statistical evaluations) leading to the results obtained from this data in the present study can be obtained from the corresponding author on request.

## Supplementary Materials

The following supporting information can be downloaded at https://www.mdpi.com/article/10.3390/ijms27219999/s1.
