# Supplementary material for: Aminosteroids from Buxaceae and Apocynaceae Plants as Antiprotozoal Agents: Structural Diversity and Structure–Activity Relationships
Source: Int J Mol Sci. 2026 Nov 8;27(21):9999. doi: 10.3390/ijms27219999 (PMC13764157; doi:10.3390/ijms27219999)
Supplement: Supplementary file 1 [file ijms-27-09999-s001.zip › ijms-4665881-supplementary.pdf]

Supplementary Materials

Aminosteroids from Buxaceae and Apocynaceae Plants as Antiprotozoal Agents: Structural Diversity and Structure–Activity Relationships

Justus W. Mukavi [1], Lizanne Schäfer [1], Lara U. Szabó [1], Charles O. Nnadi [1], Julia B. Althaus [1], Pascal Mäser [2,3] and Thomas J. Schmidt [1,*]

[1] University of Münster, Institute of Pharmaceutical Biology and Phytochemistry, PharmaCampus, Corrensstrasse 48, D-48149 Münster, Germany; justusmukavi32@gmail.com (J.W.M.); lizanne.schaefer@gmail.com (L.S.); laraszabo@web.de (L.U.S.); charles.nnadi@unn.edu.ng (C.O.N.); julia.althaus@uni-muenster.de (J.B.A.)

[2] Swiss Tropical and Public Health Institute (Swiss TPH), Kreuzstrasse 2, CH-4123 Allschwil, Switzerland; pascal.maeser@swisstph.ch

[3] University of Basel, Petersplatz 1, CH-4003 Basel, Switzerland

* Correspondence: thomschm@uni-muenster.de; Tel.: +49-251-83-33378

Table S1. Bioactivity data [-log(IC_{50}, mol/L)=pIC_{50}] values and important characteristics of the compounds under study. The structures are shown in Figure 1, main manuscript. Note that in cases with duplicate isolation (see column dupl#), the structure was included only once in the data set for 3D QSAR and mean pIC_{50} values of both determinations were used in these cases when available. #HAtm: number of heavy atoms; #AroAtm: number of aromatic heavy atoms; Fr. Csp3: Fraction of sp3 carbon atoms of all carbon atoms; #Rot.bnd: number of rotatable bonds; #HBA: number of hydrogen bond acceptors; #HBD: number of hydrogen bond donors.

Name	cpd#	dupl#	Formula	MW	pIC_{50} (Tbr)	pIC_{50} (Pf)	pIC_{50} (L6)	#HAtm	#Aro Atm	Fr. Csp3	#Rot. bnd.	#HBA	#HBD
Cyclovirobuxeine-B	1_1	2_14	C27H46N2O	414.67	5.824	5.585	4.450	30	0	0.93	3	3	2
O-Tigloylcyclovirobuxeine-B	1_2		C32H52N2O2	496.77	5.495	5.979	4.721	36	0	0.84	6	4	1
O-Tigloylcyclomicrophylline-B	1_3		C32H52N2O3	512.77	5.046	6.009	4.156	37	0	0.84	7	5	2
Cyclomicrophylline-A	1_4		C28H48N2O2	444.69	5.631	5.754	4.004	32	0	0.93	4	4	2
O-Tigloylcyclomicrophylline-A	1_5		C33H54N2O3	526.79	5.538	5.387	4.642	38	0	0.85	7	5	1
Cyclomicrophyllidine-A	1_6		C35H52N2O3	548.8	5.886	6.155	4.770	40	6	0.74	7	5	1
Cyclomicrophyllidine-B	1_7		C34H50N2O3	534.77	5.444	6.699	4.524	39	6	0.74	7	5	2
O-Benzoyl-cycloprotobuxoline-D	1_8		C33H50N2O2	506.76	5.959	6.745	4.883	37	6	0.79	6	4	2
N-Benzoyl-O-acetyl-cycloxo-buxoline-F	1_9		C35H50N2O4	562.78	5.620	5.420	4.137	41	6	0.74	8	5	1
29-Hydroxy-cyclomikuranine-L	1_10		C26H43NO3	417.62	5.155	5.658	3.977	30	0	0.96	3	4	2
N_b-dimethylcycloxobuxoviricine	1_11		C26H41NO2	399.61	5.201	5.456	4.322	29	0	0.88	2	3	1
(E)-Cyclobuxophyllinine-M	1_12		C25H39NO	369.58	5.658	5.367	4.842	27	0	0.88	1	2	1
(Z)-Cyclobuxophyllinine-M	1_13		C25H39NO	369.58	5.658	5.367	4.842	27	0	0.88	1	2	1
(E)-Cyclosuffrobuxinine-M	1_14		C24H35NO	353.54	5.678	5.509	4.532	26	0	0.79	1	2	1
(Z)-Cyclosuffrobuxinine-M	1_15		C24H35NO	353.54	5.678	5.509	4.532	26	0	0.79	1	2	1
Cyclomicrobuxinine	1_16		C24H37NO2	371.56	3.888	4.963	4.086	27	0	0.88	2	3	2
Cyclomicrobuxine	1_17		C25H39NO2	385.58	4.357	5.167	4.157	28	0	0.88	2	3	1
Irehine	1_18		C23H39NO	345.56	5.194	5.420	4.411	25	0	0.91	2	2	1
16α-hydroxybuxaminone	1_19		C26H41NO2	399.61	4.879	5.398	3.948	29	0	0.81	2	3	1
N_{20}-acetylbuxamine-E	1_20		C28H46N2O	426.68	3.907	5.137	3.889	31	0	0.82	4	2	1
N-Benzoyl-O-acetylbuxodienine-E	1_21		C35H50N2O3	546.78	5.208	5.208	4.807	40	6	0.66	7	4	1
N-Benzoyl-O-acetylbuxadine-E	1_22		C35H50N2O3	546.78	5.244	5.114	4.298	40	6	0.66	7	4	1
N_{20}-acetylbuxadine-G	1_23		C27H44N2O	412.65	5.886	5.252	4.387	30	0	0.81	4	2	2
17,20-dihydroxybuxadine-M	1_24		C25H41NO2	387.6	4.762	5.032	3.940	28	0	0.84	2	3	3
Cycloprotobuxoline-C	2_1		C27H48N2O	416.68	5.174	4.553	3.500	30	0	1	3	3	2

Cycloprotobuxoline-C N_{20}-oxide	2_2		C27H48N2O2	432.68	5.420	5.022	4.018	31	0	1	3	3	2
16α-Hydroxycycloprotobuxoline-C	2_3		C27H48N2O2	432.68	5.137	4.824	3.971	31	0	1	3	4	3
Cycloprotobuxoline-D	2_4		C26H46N2O	402.66	5.638	5.959	4.796	29	0	1	3	3	3
29-Trimethoxybenzoyloxy cycloprotobuxoline-C	2_5		C37H58N2O6	626.87	5.796	6.301	5.229	45	6	0.81	10	8	2
N_3-Demethylcycloprotobuxoline-C	2_6		C26H46N2O	402.66	6.046	5.523	4.301	29	0	1	2	3	2
16α-Hydroxy-N_3-demethylcycloprotobuxoline-C	2_7		C26H46N2O2	418.66	4.319	4.310	4.108	30	0	1	2	4	3
Cycloprotobuxoline-D N_3-*cis*-formamide	2_8b		C27H46N2O2	430.67	4.387	4.523	4.284	31	0	0.96	4	3	2
Cycloprotobuxoline-D N_3-*trans*-formamide	2_8a		C27H46N2O2	430.67	4.387	4.523	4.284	31	0	0.96	4	3	2
16α-Hydroxycycloprotobuxoline-C N_3-*cis*-formamide	2_9b		C28H48N2O3	460.69	4.409	4.585	4.168	33	0	0.96	4	4	2
16α-Hydroxycycloprotobuxoline-C N_3-*trans*-formamide	2_9a		C28H48N2O3	460.69	4.409	4.585	4.168	33	0	0.96	4	4	2
Cyclonataminol	2_10		C28H48N2O2	444.69	4.432	4.699	4.292	32	0	0.93	3	4	2
N_3-Demethyl cyclonataminol	2_11		C27H46N2O2	430.67	4.854	4.721	4.310	31	0	0.93	3	4	3
Deoxycyclovirobuxeine-B	2_12		C27H46N2	398.67	6.097	5.268	4.086	29	0	0.93	3	2	1
Cyclovirobuxeine-A	2_13		C28H48N2O	428.69	4.959	5.076	4.114	31	0	0.93	3	3	1
Cyclovirobuxeine-B	2_14	1_1	C27H46N2O	414.67	4.796	4.959	4.244	30	0	0.93	3	3	2
N_{20}-Demethyl deoxycyclobuxoxazine A	2_15		C27H46N2O	414.67	5.181	5.237	4.620	30	0	1	2	3	1
Obtusibuxeine A (16)	2_16		C24H39NO2	373.57	4.658	4.886	4.347	27	0	0.96	3	3	1
O_{10}-Obtusifuranamine-A	2_17		C35H48N2O6	592.77	4.357	4.959	4.301	43	6	0.66	7	7	3
O_{10}-Obtusifuranamine-B	2_18		C40H50N2O6	654.83	5.310	4.770	4.569	48	12	0.55	8	7	3
16-Deoxy-O_{10}-obtusifuranamine-B	2_19		C40H50N2O5	638.84	5.699	4.745	4.796	47	12	0.55	8	6	2
O_2-Natafuranamine	2_20		C35H48N2O6	592.77	4.638	5.061	4.366	43	6	0.71	7	7	2
Obtusiepoxamine-A	2_21		C35H48N2O5	576.77	5.658	5.000	4.469	42	6	0.66	7	6	2
Obtusidienolamine-A	2_22		C35H50N2O5	578.78	5.187	4.620	4.319	42	6	0.66	7	6	3
Deoxyobtusidienolamine-A	2_23		C35H50N2O4	562.78	5.187	4.523	4.409	41	6	0.66	7	5	2
Obtusiaminocyclin	2_24		C24H33NO2	367.52	4.585	5.194	4.292	27	0	0.83	0	3	1
5,6-Dehydro-desacyl-epipachysamine A	3_1		C24H42N2	358.6	5.495	5.815	4.481	26	0	0.92	3	2	1
Desacyl-epipachysamine A	3_2		C24H44N2	360.62	5.456	5.842	4.523	26	0	1	3	2	1
Pactermine A	3_3		C29H43N3O	449.67	5.466	5.936	4.991	33	6	0.72	5	3	1
Epipachysamine B	3_4		C29H45N3O	451.69	5.553	6.155	5.036	33	6	0.79	5	3	1
N_3-chloromethyl-desacyl-epipachysamine A	3_5		C25H46ClN2+	410.1	5.721	6.409	3.866	28	0	1	4	1	1
3α,4α-Diapachsanaximine A	3_6		C31H48N2O2	480.73	6.951	6.201	4.824	35	6	0.77	6	4	1

5α-Hydroxy-diapachysanaximine A	3_7		C31H48N2O3	496.72	5.301	5.638	4.796	36	6	0.77	6	5	2
Pachysamine A (8)	3_8		C24H44N2	360.62	5.886	5.510	4.752	26	0	1	3	2	1
3β-Dimethylamino-pregnan-20-on	3_9		C23H39NO	345.56	5.319	4.745	4.854	25	0	0.96	2	2	0
Pachysandrine D	3_10		C29H50N2O2	458.72	5.996	6.013	5.022	33	0	0.9	6	4	1
Terminaline	3_11		C23H41NO2	363.58	4.907	5.155	4.222	26	0	1	2	3	2
Sarcodinine	3_12		C25H44N2	372.63	5.367	5.114	4.745	27	0	0.92	3	2	0
N-Methyl-desacyl-epipachysamine A	3_13		C25H46N2	374.65	5.409	5.387	4.959	27	0	1	3	2	0
Epipachysamine A	3_14		C26H46N2O	402.66	5.383	4.444	4.638	29	0	0.96	4	2	0
Spiropachysine	3_15		C31H46N2O	462.71	5.417	5.222	4.896	34	6	0.77	2	2	0
4β-Hydroxy-hookerianamide N	3_16		C28H48N2O3	460.69	4.851	4.975	4.102	33	0	0.89	5	4	3
2β,3β,4β-Diapachysamine K	3_17		C30H46N2O3	482.7	5.004	4.097	4.553	35	6	0.77	5	4	3
3β-Dimethylamino-pregnane-20-oxime	3_18		C23H40N2O	360.58	5.124	5.180	4.395	26	0	0.96	2	3	1
3β-holaphyllamine	4_1		C21H33NO	315.49	6.396	n.d.	5.293	23	0	0.86	1	2	1
Holaphyllamineacetamide	4_2		C23H35NO2	357.53	5.316	n.d.	4.813	26	0	0.83	3	2	1
Methylholaphyllamine	4_3		C22H35NO	329.52	7.125	n.d.	5.606	24	0	0.86	2	2	1
3α-holaphyllamine	4_4		C21H33NO	315.49	6.432	n.d.	4.799	23	0	0.86	1	2	1
3β-dihydroholaphyllamine	4_5	5_18	C21H35NO	317.51	6.173	n.d.	4.760	23	0	0.95	1	2	1
3α-dihydroholaphyllamine	4_6		C21H35NO	317.51	5.919	n.d.	4.771	23	0	0.95	1	2	1
holadienine.mol	4_7	5_12	C22H31NO	325.49	4.828	n.d.	n.a.	24	0	0.77	0	2	0
holonamine.mol	4_8		C21H27NO2	325.44	5.062	n.d.	4.188	24	0	0.71	0	3	1
cona-4,6-diene-3-one	4_9		C22H31NO	325.49	4.757	n.d.	3.890	24	0	0.77	0	2	0
cona-3,5-diene-7-one	4_10		C22H31NO	325.49	5.133	n.d.	4.380	24	0	0.77	0	2	0
Conessimine	4_11		C23H38N2	342.56	6.778	n.d.	4.297	25	0	0.91	1	2	1
Isoconessimine	4_12	5_2	C23H38N2	342.56	6.778	n.d.	4.562	25	0	0.91	1	2	1
Conessine	4_13	5_3	C24H40N2	356.59	6.378	n.d.	4.213	26	0	0.92	1	2	0
Holarrhesine	4_14		C29H46N2O2	454.69	6.924	n.d.	4.844	33	0	0.83	5	4	1
Holarrhetine	4_15		C30H48N2O2	468.71	5.781	n.d.	4.010	34	0	0.83	5	4	0
Holarrheline	4_16		C23H38N2O	358.56	5.086	n.d.	3.743	26	0	0.91	1	3	2
Holarrhenine	4_17		C24H40N2O	372.59	4.680	n.d.	3.904	27	0	0.92	1	3	1
3β-Aminoconamine	5_1		C22H36N2	328.53	5.886	5.770	4.523	24	0	0.91	0	2	1
Isoconessimine	5_2	4_12	C23H38N2	342.56	6.301	5.699	4.456	25	0	0.91	1	2	1

Conessine	5_3	4_13	C24H40N2	356.59	6.699	5.699	4.602	26	0	0.92	1	2	0
3α-Aminoconamine	5_4		C22H36N2	328.53	4.886	5.824	4.523	24	0	0.91	0	2	1
3β-Aminoconanine	5_5		C22H38N2	330.55	5.377	5.854	4.699	24	0	1	0	2	1
Dihydroisoconessine	5_6		C23H40N2	344.58	5.398	5.699	4.585	25	0	1	1	2	1
3α-Aminoconanine	5_7		C22H38N2	330.55	4.357	6.000	4.638	24	0	1	0	2	1
N_3-Chloromethylconessine	5_8		C25H42ClN2+	406.07	4.770	4.921	n.a.	28	0	0.92	2	1	0
N_3-Formylamino- dihydroisoconessine	5_9		C23H38N2O	358.56	5.292	6.155	4.523	26	0	0.96	2	2	1
Irehline	5_10		C21H32N2	312.49	5.538	5.482	4.495	23	0	0.86	0	2	1
Wrightiamine A	5_11		C21H34N2	314.51	5.824	5.745	4.796	23	0	0.95	0	2	1
Holadienine	5_12	4_7	C22H31NO	325.49	4.796	5.319	4.244	24	0	0.77	0	2	0
3β-Amino-5α*H*-pregnan-20α-ol	5_13		C21H37NO	319.52	5.699	4.886	4.745	23	0	1	1	2	2
3α-Amino-5-pregnen-20α-ol	5_14		C21H35NO	317.51	4.959	4.854	4.620	23	0	0.9	1	2	2
3α-Amino-5α*H*-pregnan-20α-ol (Funtumidine)	5_15		C21H37NO	319.52	5.745	5.149	4.721	23	0	1	1	2	2
20α-Amino-5-pregnen-3β-ol (Holafebrine)	5_16		C21H35NO	317.51	5.276	4.959	4.886	23	0	0.9	1	2	2
20α-Aminopregnan-3β-ol	5_17		C21H37NO	319.52	5.244	5.143	4.886	23	0	1	1	2	2
3β-Dihydroholaphyllamine	5_18	4_5	C21H35NO	317.51	6.699	5.310	5.347	23	0	0.95	1	2	1
20α-Amino-5-pregnene-3β,18-diol	5_19		C21H35NO2	333.51	4.721	4.796	4.284	24	0	0.9	2	3	3
20α-Amino-5α-pregnane-3β,18-diol	5_20		C21H37NO2	335.52	4.745	4.959	4.319	24	0	1	2	3	3

n.d. not determined; n.a. not accessible, IC_{50} >100 µmol/L; pIC_{50} cutoff value 3.5 used instead.

Table S2. Predicted physicochemical and ADME data of the compounds under study as obtained from the SwissADME web tool, part 1.
MR: Molar refractivity; TPSA: total polar surface area; Cons. Log P: Consensus (mean) of octanol/water coefficients (log P values) predicted by five different methods; Cons. LogS: Consensus (mean) of log solubility values predicted by three different methods; GI abs.: Gastro-intestinal absorption; BBB perm.: Blood-brain-barrier permeation; CYP …inh. Cytochrome P450 enzyme inhibitor. For definitions of all calculated characteristics and their original literature see the SwissADME website [14].

Name	cpd#	dupl#	MR	TPSA	Cons. Log P	Cons. LogS	GI abs.	BBB perm.	Pgp subs.	CYP1A2 inh.	CYP2C19 inh.	CYP2C9 inh.	CYP2D6 inh.	CYP3A4 inh.
Cyclovirobuxeine-B	1_1	2_14	126.42	35.5	4.68	-5.73	High	Yes	No	No	No	No	No	No
O-Tigloylcyclovirobuxeine-B	1_2		150.1	41.57	5.74	-6.91	High	No	No	No	No	No	No	No
O-Tigloylcyclomicrophylline-B	1_3		151.26	61.8	5	-6.19	High	Yes	Yes	No	No	No	No	No
Cyclomicrophylline-A	1_4		132.48	46.94	4.01	-4.82	High	Yes	Yes	No	No	No	No	No
O-Tigloylcyclomicrophylline-A	1_5		156.17	53.01	5.26	-6.30	High	Yes	Yes	No	No	No	No	No
Cyclomicrophyllidine-A	1_6		162.12	53.01	5.47	-7.19	High	No	Yes	No	No	No	No	No
Cyclomicrophyllidine-B	1_7		157.22	61.8	5.22	-7.07	High	No	Yes	No	No	No	No	No
O-Benzoyl-cycloprotobuxoline-D	1_8		151.63	50.36	6.16	-8.29	High	No	No	No	No	No	No	No
N-Benzoyl-O-acetyl-cycloxo-buxoline-F	1_9		161.74	75.71	5.41	-7.39	High	No	Yes	No	No	No	No	Yes
29-Hydroxy-cyclomikuranine-L	1_10		120.65	60.77	3.92	-5.14	High	Yes	Yes	No	No	No	No	No
N_b-dimethylcycloxobuxoviricine	1_11		119.01	40.54	4.48	-5.49	High	Yes	No	No	No	No	No	No
(E)-Cyclobuxophyllinine-M	1_12		112.95	29.1	5.2	-6.16	High	Yes	No	No	No	No	No	No
(Z)-Cyclobuxophyllinine-M	1_13		112.95	29.1	5.2	-6.16	High	Yes	No	No	No	No	No	No
(E)-Cyclosuffrobuxinine-M	1_14		107.93	29.1	4.71	-5.35	High	Yes	No	No	No	No	No	No
(Z)-Cyclosuffrobuxinine-M	1_15		107.93	29.1	4.72	-5.35	High	Yes	No	No	No	No	No	No
Cyclomicrobuxinine	1_16		109.56	49.33	3.98	-4.87	High	Yes	Yes	No	No	No	No	No
Cyclomicrobuxine	1_17		114.46	40.54	4.17	-4.98	High	Yes	No	No	No	No	No	No
Irehine	1_18		107.28	23.47	4.49	-4.85	High	Yes	No	No	No	No	Yes	No
16α-hydroxybuxaminone	1_19		121.17	40.54	4.35	-5.00	High	Yes	No	No	No	No	No	No
N_{20}-acetylbuxamine-E	1_20		132.42	32.34	5.15	-5.88	High	No	No	No	No	No	No	No
N-Benzoyl-O-acetylbuxodienine-E	1_21		163.22	58.64	5.76	-7.50	High	No	Yes	No	No	No	No	Yes
N-Benzoyl-O-acetylbuxadine-E	1_22		163.22	58.64	5.74	-7.46	High	No	Yes	No	No	No	No	Yes
N_{20}-acetylbuxadine-G	1_23		127.52	41.13	4.92	-5.74	High	Yes	No	No	No	No	No	No
17,20-dihydroxybuxadine-M	1_24		117.27	52.49	4.15	-4.69	High	Yes	Yes	No	No	No	No	No
Cycloprotobuxoline-C	2_1		126.89	35.5	4.89	-6.04	High	Yes	No	No	No	No	No	No

Cycloprotobuxoline-C N_{20}-oxide	2_2		130.26	61.69	3.54	-6.04	High	Yes	Yes	No	No	No	No	No
16α-Hydroxycycloprotobuxoline-C	2_3		128.05	55.73	4.06	-5.30	High	Yes	Yes	No	No	No	No	No
Cycloprotobuxoline-D	2_4		121.99	44.29	4.66	-5.93	High	Yes	Yes	No	No	No	No	No
29-Trimethoxybenzoyloxy cycloprotobuxoline-C	2_5		177.17	89.49	5.59	-8.04	High	No	Yes	No	No	No	No	No
N_3-Demethylcycloprotobuxoline-C	2_6		121.99	49.49	4.48	-5.59	High	Yes	Yes	No	No	No	No	No
16α-Hydroxy-N_3-demethylcycloprotobuxoline-C	2_7		123.15	69.72	3.71	-4.84	High	Yes	Yes	No	No	No	No	No
Cycloprotobuxoline-D N_3-*cis*-formamide	2_8b		127.48	52.57	4.51	-5.72	High	Yes	Yes	No	No	No	No	No
Cycloprotobuxoline-D N_3-*trans*-formamide	2_8a		127.48	52.57	4.51	-5.72	High	Yes	Yes	No	No	No	No	No
16α-Hydroxycycloprotobuxoline-C N_3-*cis*-formamide	2_9b		133.54	64.01	3.92	-5.10	High	Yes	Yes	No	No	No	No	No
16α-Hydroxycycloprotobuxoline-C N_3-*trans*-formamide	2_9a		133.54	64.01	3.92	-5.10	High	Yes	Yes	No	No	No	No	No
Cyclonataminol	2_10		132.48	46.94	4	-4.90	High	Yes	Yes	No	No	No	No	No
N_3-Demethyl cyclonataminol	2_11		127.58	55.73	3.72	-4.79	High	Yes	Yes	No	No	No	No	No
Deoxycyclovirobuxeine-B	2_12		125.26	15.27	5.4	-6.17	High	No	No	No	No	No	No	No
Cyclovirobuxeine-A	2_13		131.32	26.71	4.79	-5.54	High	Yes	No	No	No	No	No	No
Cyclovirobuxeine-B	2_14	1_1	126.42	35.5	4.68	-5.73	High	Yes	No	No	No	No	No	No
N_{20}-Demethyl deoxycyclobuxoxazine A	2_15		128.62	24.5	5.09	-6.18	High	Yes	No	No	No	No	No	No
Obtusibuxeine A (16)	2_16		109.96	38.33	4.26	-5.05	High	Yes	No	No	No	No	No	No
O_{10}-Obtusifuranamine-A	2_17		164.56	108.33	3.43	-5.27	High	No	Yes	No	No	No	No	No
O_{10}-Obtusifuranamine-B	2_18		184.46	108.33	4.44	-7.00	High	No	Yes	No	No	No	No	No
16-Deoxy-O_{10}-obtusifuranamine-B	2_19		183.3	88.1	5.38	-7.75	High	No	Yes	No	No	No	No	No
O_2-Natafuranamine	2_20		162.84	100.63	3.73	-5.59	High	No	Yes	No	No	No	No	No
Obtusiepoxamine-A	2_21		163.39	88.1	4.25	-6.01	High	No	Yes	No	No	No	No	Yes
Obtusidienolamine-A	2_22		165.55	99.1	4.15	-5.97	High	No	Yes	No	No	No	No	Yes
Deoxyobtusidienolamine-A	2_23		164.39	78.87	4.95	-6.72	High	No	Yes	No	No	No	No	Yes
Obtusiaminocyclin	2_24		110.44	46.17	3.77	-4.63	High	Yes	No	No	No	No	No	No
5,6-Dehydro-desacyl-epipachysamine A	3_1		113.73	15.27	4.73	-5.17	High	Yes	No	No	No	No	Yes	No
Desacyl-epipachysamine A	3_2		114.2	15.27	4.96	-5.64	High	Yes	No	No	No	No	Yes	No

Pactermine A	3_3		136.43	45.23	4.88	-6.30	High	Yes	No	No	No	No	No	No
Epipachysamine B	3_4		136.91	45.23	5.12	-6.77	High	Yes	No	No	No	No	No	No
N_3-chloromethyl-desacyl-epipachysamine A	3_5		123.29	12.03	3.52	-6.65	Low	No	Yes	No	No	No	No	No
3α,4α-Diapachsanaximine A	3_6		145	41.57	5.68	-7.27	High	No	No	No	No	No	No	No
5α-Hydroxy-diapachysanaximine A	3_7		146.2	61.8	4.83	-6.62	High	Yes	Yes	No	No	No	No	No
Pachysamine A (8)	3_8		114.2	15.27	4.95	-5.64	High	Yes	No	No	No	No	Yes	No
3β-Dimethylamino-pregnan-20-on	3_9		106.8	20.31	4.67	-5.17	High	Yes	No	No	No	No	Yes	No
Pachysandrine D	3_10		139.05	41.57	5.39	-6.52	High	Yes	No	No	No	No	No	No
Terminaline	3_11		108.92	43.7	3.87	-4.47	High	Yes	Yes	No	No	No	No	No
Sarcodinine	3_12		118.63	6.48	4.96	-5.29	Low	No	No	No	No	No	Yes	No
N-Methyl-desacyl-epipachysamine A	3_13		119.11	6.48	5.17	-5.75	Low	No	No	No	No	No	Yes	No
Epipachysamine A	3_14		124.11	23.55	4.91	-5.58	High	Yes	No	No	No	No	Yes	No
Spiropachysine	3_15		145.86	23.55	5.76	-7.19	High	No	No	Yes	Yes	No	No	No
4β-Hydroxy-hookerianamide N	3_16		135.48	72.8	3.99	-5.26	High	Yes	Yes	No	No	No	No	No
2β,3β,4β-Diapachysamine K	3_17		141.44	72.8	4.26	-6.00	High	Yes	Yes	No	No	No	No	No
3β-Dimethylamino-pregnane-20-oxime	3_18		111.01	35.83	4.71	-5.30	High	Yes	No	No	No	No	No	No
3β-holaphyllamine	4_1		96.52	43.09	3.87	-4.23	High	Yes	No	No	No	No	No	No
Holaphyllamineacetamide	4_2		106.43	46.17	4.05	-4.68	High	Yes	No	No	No	No	No	No
Methylholaphyllamine	4_3		101.42	29.1	4.24	-4.69	High	Yes	No	No	No	No	No	No
3α-holaphyllamine	4_4		96.52	43.09	3.88	-4.23	High	Yes	No	No	No	No	No	No
3β-dihydroholaphyllamine	4_5	5_18	96.99	43.09	4.04	-4.60	High	Yes	No	No	No	No	No	No
3α-dihydroholaphyllamine	4_6		96.99	43.09	4.07	-4.60	High	Yes	No	No	No	No	No	No
holadienine.mol	4_7	5_12	102.84	20.31	3.84	-4.21	High	Yes	No	No	No	Yes	No	No
holonamine.mol	4_8		99.4	49.66	2.8	-2.98	High	Yes	Yes	No	No	No	No	No
cona-4,6-diene-3-one	4_9		102.84	20.31	3.75	-3.97	High	Yes	No	No	No	Yes	No	No
cona-3,5-diene-7-one	4_10		102.84	20.31	3.83	-4.21	High	Yes	No	No	No	Yes	No	No
Conessimine	4_11		110.72	15.27	4.1	-4.49	High	Yes	No	No	No	No	No	No
Isoconessimine	4_12	5_2	110.72	15.27	4.11	-4.49	High	Yes	No	No	No	No	No	No
Conessine	4_13	5_3	115.63	6.48	4.36	-4.61	High	Yes	No	No	No	No	No	No
Holarrhesine	4_14		140.38	41.57	4.8	-5.60	High	Yes	No	No	No	No	No	No

Holarrhetine	4_15		145.28	32.78	5.06	-5.71	High	Yes	No	No	No	No	No	No
Holarrheline	4_16		111.89	35.5	3.24	-3.75	High	Yes	Yes	No	No	No	No	No
Holarrhenine	4_17		116.79	26.71	3.5	-3.87	High	Yes	No	No	No	No	No	No
3β-Aminoconamine	5_1		105.82	29.26	3.75	-4.04	High	Yes	No	No	No	No	No	No
Isoconessimine	5_2	4_12	110.72	15.27	4.11	-4.49	High	Yes	No	No	No	No	No	No
Conessine	5_3	4_13	115.63	6.48	4.36	-4.61	High	Yes	No	No	No	No	No	No
3α-Aminoconamine	5_4		105.82	29.26	3.75	-4.04	High	Yes	No	No	No	No	No	No
3β-Aminoconanine	5_5		106.3	29.26	3.96	-4.50	High	Yes	No	Yes	No	No	No	No
Dihydroisoconessine	5_6		111.2	15.27	4.33	-4.96	High	Yes	No	No	No	No	No	No
3α-Aminoconanine	5_7		106.3	29.26	3.97	-4.50	High	Yes	No	Yes	No	No	No	No
N_3-Chloromethylconessine	5_8		124.71	3.24	2.9	-5.63	Low	No	Yes	No	No	No	No	No
N_3-Formylamino-dihydroisoconessine	5_9		111.79	32.34	3.96	-4.80	High	Yes	No	No	No	No	No	No
Irehline	5_10		101.22	38.38	3.57	-3.55	High	Yes	Yes	No	No	No	No	No
Wrightiamine A	5_11		101.69	38.38	3.79	-4.02	High	Yes	Yes	No	No	No	No	No
Holadienine	5_12	4_7	102.84	20.31	3.84	-4.21	High	Yes	No	No	No	Yes	No	No
3β-Amino-5α*H*-pregnan-20α-ol	5_13		97.95	46.25	4.05	-4.64	High	Yes	Yes	No	No	No	No	No
3α-Amino-5-pregnen-20α-ol	5_14		97.48	46.25	3.88	-4.28	High	Yes	Yes	No	No	No	No	No
3α-Amino-5α*H*-pregnan-20α-ol (Funtumidine)	5_15		97.95	46.25	4.08	-4.64	High	Yes	Yes	No	No	No	No	No
20α-Amino-5-pregnen-3β-ol (Holafebrine)	5_16		97.48	46.25	3.87	-4.28	High	Yes	Yes	No	No	No	No	No
20α-Aminopregnan-3β-ol	5_17		97.95	46.25	4.06	-4.64	High	Yes	Yes	No	No	No	No	No
3β-Dihydroholaphyllamine	5_18	4_5	96.99	43.09	4.04	-4.60	High	Yes	No	No	No	No	No	No
20α-Amino-5-pregnene-3β,18-diol	5_19		98.64	66.48	3.01	-3.36	High	Yes	Yes	No	No	No	No	No
20α-Amino-5α-pregnane-3β,18-diol	5_20		99.12	66.48	3.31	-3.83	High	Yes	Yes	No	No	No	No	No

Table S3. Predicted physicochemical and ADME data of the compounds under study as obtained from the SwissADME web tool, part 2.
logKp: Skin permeation coefficient; #viol. : number of violations of various druglikeness-schemes; Bioav. Score: Bioavailablity Score; PAINS #alerts: Number of pan-assay-interference alerts; Brenk #alerts: Structural alerts; Leadl. #vilol.: Violations of leadlikeness; Synth. Acc.; Synthetic accessibility. For definitions of all calculated characteristics and their original literature see the SwissADME website [14].

Name	cpd#	dupl#	log Kp (cm/s)	Lipinski #viol.	Ghose #viol.	Veber #viol.	Egan #viol.	Muegge #viol.	Bioav. Score	PAINS #alerts	Brenk #alerts	Leadl. #viol.	Synth. Acc.
Cyclovirobuxeine-B	1_1	2_14	-4.7	1	1	0	0	1	0.55	0	1	2	6.63
O-Tigloylcyclovirobuxeine-B	1_2		-4.4	1	4	0	1	1	0.55	0	2	2	7.2
O-Tigloylcyclomicrophylline-B	1_3		-5.3	2	3	0	0	1	0.17	0	2	2	7.33
Cyclomicrophylline-A	1_4		-5.8	0	2	0	0	0	0.55	0	1	2	6.89
O-Tigloylcyclomicrophylline-A	1_5		-5.1	2	3	0	0	1	0.17	0	2	2	7.45
Cyclomicrophyllidine-A	1_6		-4.9	2	4	0	1	1	0.17	0	1	2	7.13
Cyclomicrophyllidine-B	1_7		-5.1	2	3	0	0	1	0.17	0	1	2	7.01
O-Benzoyl-cycloprotobuxoline-D	1_8		-3.9	2	4	0	1	1	0.17	0	0	2	6.35
N-Benzoyl-O-acetyl-cycloxo-buxoline-F	1_9		-5.5	2	4	0	1	1	0.17	0	0	3	6.37
29-Hydroxy-cyclomikuranine-L	1_10		-5.6	0	1	0	0	0	0.55	0	0	2	5.64
N_b-dimethylcycloxobuxoviricine	1_11		-4.9	1	0	0	0	1	0.55	0	0	2	6.08
(E)-Cyclobuxophyllinine-M	1_12		-4.4	1	0	0	0	1	0.55	0	1	2	5.54
(Z)-Cyclobuxophyllinine-M	1_13		-4.4	1	0	0	0	1	0.55	0	1	2	5.54
(E)-Cyclosuffrobuxinine-M	1_14		-5.1	1	0	0	0	0	0.55	0	2	2	5.4
(Z)-Cyclosuffrobuxinine-M	1_15		-5.1	1	0	0	0	0	0.55	0	2	2	5.4
Cyclomicrobuxinine	1_16		-5.6	0	0	0	0	0	0.55	0	1	2	5.31
Cyclomicrobuxine	1_17		-5.3	0	0	0	0	0	0.55	0	1	2	5.43
Irehine	1_18		-4.7	1	0	0	0	1	0.55	0	1	1	5.11
16α-hydroxybuxaminone	1_19		-5.4	0	0	0	0	0	0.55	0	0	2	5.63
N_{20}-acetylbuxamine-E	1_20		-4.9	1	3	0	1	1	0.55	0	0	2	5.79
N-Benzoyl-O-acetylbuxodienine-E	1_21		-4.9	2	4	0	1	1	0.17	0	0	2	6.1
N-Benzoyl-O-acetylbuxadine-E	1_22		-4.9	2	4	0	1	1	0.17	0	1	2	6.01
N_{20}-acetylbuxadine-G	1_23		-5.2	1	2	0	0	1	0.55	0	1	2	5.52
17,20-dihydroxybuxadine-M	1_24		-5.9	0	0	0	0	0	0.55	0	1	2	5.55

Cycloprotobuxoline-C	2_1		-4.6	1	1	0	0	1	0.55	0	0	2	5.73
Cycloprotobuxoline-C N_{20}-oxide	2_2		-5	0	2	0	0	1	0.55	0	2	2	6.06
16α-Hydroxycycloprotobuxoline-C	2_3		-5.5	0	1	0	0	0	0.55	0	0	2	5.93
Cycloprotobuxoline-D	2_4		-4.8	1	1	0	0	1	0.55	0	0	2	5.61
29-Trimethoxybenzoyloxy cycloprotobuxoline-C	2_5		-5.2	1	4	0	0	2	0.55	0	0	3	7.46
N_3-Demethylcycloprotobuxoline-C	2_6		-4.8	1	1	0	0	1	0.55	0	0	2	5.57
16α-Hydroxy-N_3-demethylcycloprotobuxoline-C	2_7		-5.8	0	1	0	0	0	0.55	0	0	2	5.78
Cycloprotobuxoline-D N_3-*cis*-formamide	2_8b		-5	1	1	0	0	1	0.55	0	1	2	5.66
Cycloprotobuxoline-D N_3-*trans*-formamide	2_8a		-5	1	1	0	0	1	0.55	0	1	2	5.66
16α-Hydroxycycloprotobuxoline-C N_3-*cis*-formamide	2_9b		-5.7	0	2	0	0	0	0.55	0	1	2	5.97
16α-Hydroxycycloprotobuxoline-C N_3-*trans*-formamide	2_9a		-5.7	0	2	0	0	0	0.55	0	1	2	5.97
Cyclonataminol	2_10		-5.7	0	2	0	0	0	0.55	0	1	2	6.85
N_3-Demethyl cyclonataminol	2_11		-5.9	0	1	0	0	0	0.55	0	1	2	6.73
Deoxycyclovirobuxeine-B	2_12		-4.2	1	2	0	0	1	0.55	0	1	2	6.6
Cyclovirobuxeine-A	2_13		-4.9	1	2	0	0	1	0.55	0	1	2	6.75
Cyclovirobuxeine-B	2_14	1_1	-4.7	1	1	0	0	1	0.55	0	1	2	6.63
N_{20}-Demethyl deoxycyclobuxoxazine A	2_15		-4.4	1	1	0	0	1	0.55	0	0	2	6.62
Obtusibuxeine A (16)	2_16		-5.4	0	0	0	0	0	0.55	0	0	2	5.35
O_{10}-Obtusifuranamine-A	2_17		-7.6	1	3	0	0	0	0.55	0	1	1	7.43
O_{10}-Obtusifuranamine-B	2_18		-6.8	1	3	0	0	1	0.55	0	1	3	7.71
16-Deoxy-O_{10}-obtusifuranamine-B	2_19		-5.9	2	4	0	1	2	0.17	0	1	3	7.62
O_2-Natafuranamine	2_20		-7.4	1	3	0	0	0	0.55	0	2	2	7.06
Obtusiepoxamine-A	2_21		-6.6	1	3	0	0	0	0.55	0	1	2	7.09
Obtusidienolamine-A	2_22		-6.8	1	3	0	0	0	0.55	0	1	2	6.46
Deoxyobtusidienolamine-A	2_23		-5.8	2	4	0	0	1	0.17	0	1	2	6.32
Obtusiaminocyclin	2_24		-6.2	0	0	0	0	0	0.55	0	1	1	5.88
5,6-Dehydro-desacyl-epipachysamine A	3_1		-4.7	1	0	0	0	1	0.55	0	1	2	5.59
Desacyl-epipachysamine A	3_2		-4.1	1	0	0	0	1	0.55	0	0	2	4.43

Pactermine A	3_3		-5	1	3	0	0	1	0.55	0	1	2	5.33
Epipachysamine B	3_4		-4.5	1	3	0	0	1	0.55	0	0	2	4.83
N_3-chloromethyl-desacyl-epipachysamine A	3_5		-3.9	0	2	0	1	1	0.55	0	3	2	5.14
3α,4α-Diapachsanaximine A	3_6		-4.1	1	4	0	1	1	0.55	0	0	2	5.43
5α-Hydroxy-diapachysanaximine A	3_7		-5.1	1	3	0	0	1	0.55	0	0	2	5.65
Pachysamine A (8)	3_8		-4.1	1	0	0	0	1	0.55	0	0	2	4.43
3β-Dimethylamino-pregnan-20-on	3_9		-4.5	1	0	0	0	1	0.55	0	0	1	4.16
Pachysandrine D	3_10		-4.1	1	3	0	0	1	0.55	0	1	2	5.77
Terminaline	3_11		-5.2	0	0	0	0	0	0.55	0	0	2	4.5
Sarcodinine	3_12		-4.5	1	1	0	0	1	0.55	0	1	2	5.71
N-Methyl-desacyl-epipachysamine A	3_13		-3.9	1	1	0	0	1	0.55	0	0	2	4.55
Epipachysamine A	3_14		-4.4	1	1	0	0	1	0.55	0	0	2	4.52
Spiropachysine	3_15		-4.1	1	3	0	1	1	0.55	0	0	2	5.62
4β-Hydroxy-hookerianamide N	3_16		-5.4	0	2	0	0	1	0.55	0	1	2	5.68
2β,3β,4β-Diapachysamine K	3_17		-5.4	0	3	0	0	1	0.55	0	0	2	5.02
3β-Dimethylamino-pregnane-20-oxime	3_18		-4.4	1	0	0	0	1	0.55	0	3	2	5.16
3β-holaphyllamine	4_1		-5.4	0	0	0	0	0	0.55	0	1	1	5.01
Holaphyllamineacetamide	4_2		-5.5	0	0	0	0	0	0.55	0	1	2	5.03
Methylholaphyllamine	4_3		-5.1	1	0	0	0	0	0.55	0	1	1	5.02
3α-holaphyllamine	4_4		-5.4	0	0	0	0	0	0.55	0	1	1	5.01
3β-dihydroholaphyllamine	4_5	5_18	-5	1	0	0	0	0	0.55	0	0	1	4.07
3α-dihydroholaphyllamine	4_6		-5	1	0	0	0	0	0.55	0	0	1	4.07
holadienine.mol	4_7	5_12	-5.2	1	0	0	0	0	0.55	0	0	1	5.29
holonamine.mol	4_8		-6.8	0	0	0	0	0	0.55	0	0	0	5.71
cona-4,6-diene-3-one	4_9		-5.5	1	0	0	0	0	0.55	0	0	1	5.38
cona-3,5-diene-7-one	4_10		-5.2	1	0	0	0	0	0.55	0	0	1	5.38
Conessimine	4_11		-5.3	1	0	0	0	0	0.55	0	1	1	5.73
Isoconessimine	4_12	5_2	-5.3	1	0	0	0	0	0.55	0	1	1	5.73
Conessine	4_13	5_3	-5	1	0	0	0	0	0.55	0	1	2	5.84
Holarrhesine	4_14		-5.2	1	2	0	0	1	0.55	0	1	2	6.57
Holarrhetine	4_15		-5	1	2	0	0	1	0.55	0	1	2	6.69

Holarrheline	4_16		-6.2	0	0	0	0	0	0.55	0	1	1	5.83
Holarrhenine	4_17		-6	0	0	0	0	0	0.55	0	1	2	5.95
3β-Aminoconamine	5_1		-5.6	1	0	0	0	0	0.55	0	1	1	5.52
Isoconessimine	5_2	4_12	-5.3	1	0	0	0	0	0.55	0	1	1	5.73
Conessine	5_3	4_13	-5	1	0	0	0	0	0.55	0	1	2	5.84
3α-Aminoconamine	5_4		-5.6	1	0	0	0	0	0.55	0	1	1	5.52
3β-Aminoconanine	5_5		-5	1	0	0	0	0	0.55	0	0	1	4.61
Dihydroisoconessine	5_6		-4.7	1	0	0	0	1	0.55	0	0	1	4.73
3α-Aminoconanine	5_7		-5	1	0	0	0	0	0.55	0	0	1	4.61
N_3-Chloromethylconessine	5_8		-4.8	0	0	0	0	1	0.55	0	4	2	6.16
N_3-Formylamino- dihydroisoconessine	5_9		-5	1	0	0	0	0	0.55	0	1	2	4.63
Irehline	5_10		-6.2	0	0	0	0	0	0.55	0	1	0	5.97
Wrightiamine A	5_11		-5.6	1	0	0	0	0	0.55	0	0	1	5.88
Holadienine	5_12	4_7	-5.2	1	0	0	0	0	0.55	0	0	1	5.29
3β-Amino-5α*H*-pregnan-20α-ol	5_13		-4.8	1	0	0	0	0	0.55	0	0	1	4.19
3α-Amino-5-pregnen-20α-ol	5_14		-5.2	1	0	0	0	0	0.55	0	1	1	5.15
3α-Amino-5α*H*-pregnan-20α-ol (Funtumidine)	5_15		-4.8	1	0	0	0	0	0.55	0	0	1	4.19
20α-Amino-5-pregnen-3β-ol (Holafebrine)	5_16		-5.2	1	0	0	0	0	0.55	0	1	1	5.07
20α-Aminopregnan-3β-ol	5_17		-4.8	1	0	0	0	0	0.55	0	0	1	4.19
3β-Dihydroholaphyllamine	5_18	4_5	-5	1	0	0	0	0	0.55	0	0	1	4.07
20α-Amino-5-pregnene-3β,18-diol	5_19		-6.4	0	0	0	0	0	0.55	0	1	0	5.13
20α-Amino-5α-pregnane-3β,18-diol	5_20		-5.9	0	0	0	0	0	0.55	0	0	1	4.32

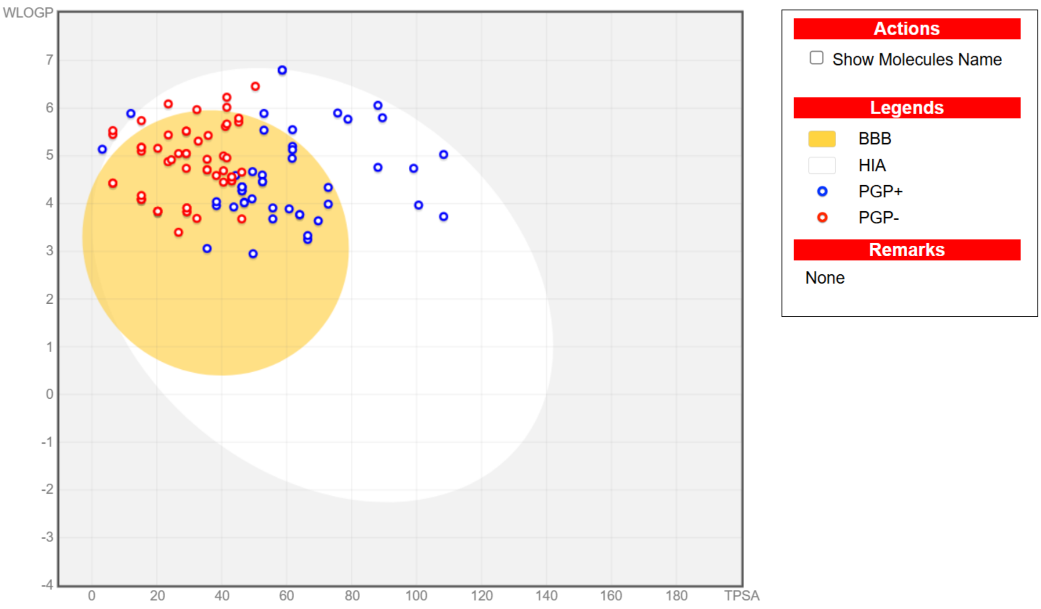

Figure S1. BOILED-egg plot of the aminosteroids under study, as obtained from the Swiss ADME web tool [16]. Compounds located within the white area are predicted to be passively absorbed in the GI after oral administration (“HIA”). Compounds located within the yellow area are predicted to pass the blood-brain barrier (“BBB”).

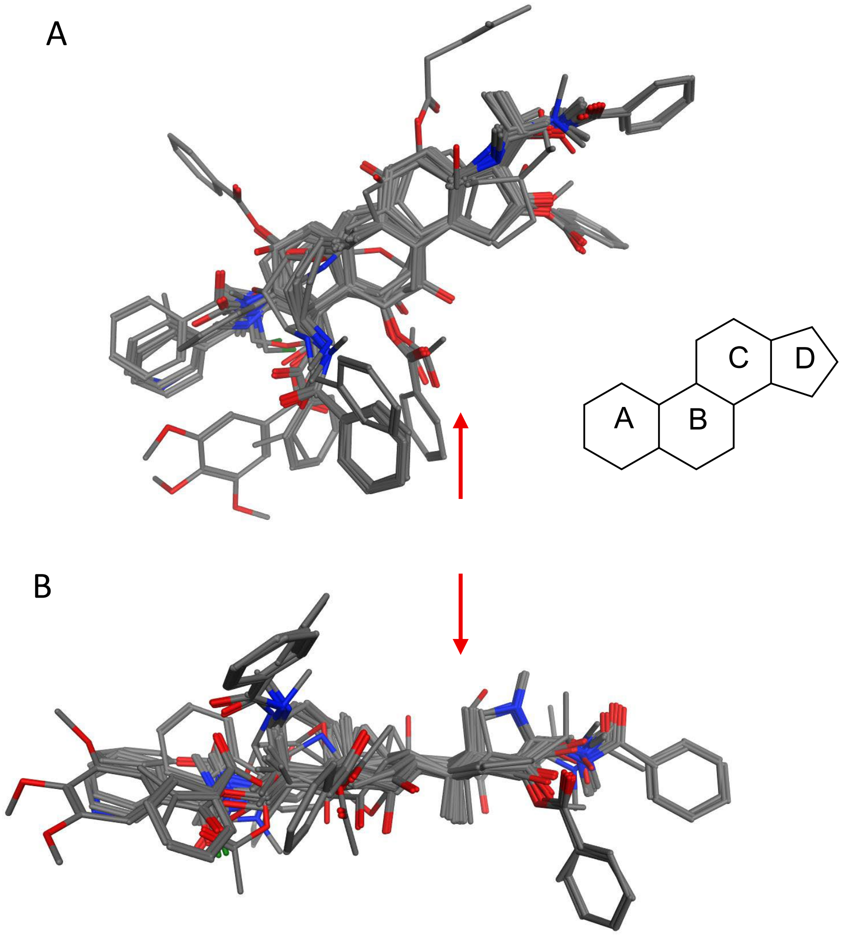

Figure S2. Molecular models of all 100 aminosteroids after alignment using the "flexalign" function of MOE [19]. The alignment is shown from two different viewing angles: A – "Top view" on the steroid cores; the 2D structure of the sterane core is shown for easier comparison. B – "Side view" rotated by approximately 90°. The red arrows indicate the viewing direction in the respective other panel.

Program Execution Code S1. Open3DQSAR commands used for model generation

The best model for anti-*Tbr* activity with 63 AS (model *Tbr*_63_t6 in Table 3 of main document) was chosen as example.

Commands and default parameters: normal black font; non-executed explanations are *written between asterisks*, input and output filenames in blue.

to start program open3dqsar.exe in a command line shell

file import, y variable definition

import type=SDF file=63_Tbr_4PH4.sdf y_var_name=pIC50(Tbr$)

grid definition, field calculation and variable pretreatment

box step=1.0

calc_field type=VDW

calc_field type=MM_ELE

cutoff field_list=1 type=max level=1.0e04

exclude type=ANY ref_field=1 field_list=2

cutoff type=max level=30.0

cutoff type=min level=-30.0

zero type=all level=0.05

test set definition: here 25%=16 compounds based on DIVPRIO$ 4PH4

set id_list=48,49,50,51,52,53,54,55,56,57,58,59,60,61,62,63 attribute=testset

removal of level variables

sdcut level=0.1

nlevel

remove_x_vars type=nlevel

equal weighting of fields

scale_x_vars type=buw

PLS regression, LOO CV, test set preditcion & output of results in sdf files (1)

pls pc=5 file=63_Tbr_4PH4_pls_01.sdf

cv pc=5 type=loo file=63_Tbr_4PH4_loo_01.sdf

predict file=63_Tbr_4PH4_ext_pred_01.sdf

selection of most significant variables and removal of less significant variables

srd pc=5 collapse=yes critical_distance=1.0 \

collapse_distance=2.0 type=weights

ffdsel pc=5 type=loo percent_dummies=20 use_srd_groups=yes \

combination_variable_ratio=1.0 fold_over=no

remove_x_vars type=ffdsel

PLS regression, LOO CV, test set preditcion & output of results in sdf files (2)

pls pc=5 file=63_Tbr_4PH4_pls_02.sdf

cv pc=5 type=loo file=63_Tbr_4PH4_loo_02.sdf

predict file=63_Tbr_4PH4_ext_pred_02.sdf

progressive scrambling

scramble pc=5 type=LOO scramblings=20 fit_order=2 critical_point=0.85

export of PLS coefficients for models with pv=n variables for visualization in MOE; in the chosen example, n=5

export type=coefficients pc=2 file=63_Tbr_4PH4_ffdsel_coefficients_2pc \

format=MOE interpolate=3

export type=coefficients pc=3 file=63_Tbr_4PH4_ffdsel_coefficients_3pc \

format=MOE interpolate=3

export type=coefficients pc=4 file=63_Tbr_4PH4_ffdsel_coefficients_4pc \

format=MOE interpolate=3

export type=coefficients pc=5 file=63_Tbr_4PH4_ffdsel_coefficients_5pc \

format=MOE interpolate=3

end program

exit

Table S4. Prediction of anti-*Pf* activity with model ***Pf*_82_t2** for untested compounds. The 3D models of these compounds' structure were obtained by applying the structural modifications to the previously aligned models of the mentioned compounds of the original data set (compare Figure 1, main publication). The resulting structures were then energy-minimized and submitted to open3dqsar for activity prediction without further changes.

1. Hybrids of **1-7** and **2-5**

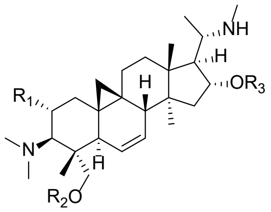

	R_1	R_2	R_3	pred. pIC_{50} (*Pf*)
new_AS_01	H	Bz(OMe)3	Bz	7.1324
new_AS_02	H	Bz	Bz	6.8293
new_AS_03	OH	Bz(OMe)3	Bz	7.2686
new_AS_04	OH	Bz	Bz	6.9656
new_AS_05	H	Bz(OMe)3	Bz(OMe)3	7.1267
new_AS_06	OH	Bz(OMe)3	Bz(OMe)3	7.2628

Bz = Benzoyl 3,4,5-Trimethoxybenzoyl
Bz(OMe)3

2. Derivatives of normal steroid **3-6** with additional aryl ester at C-16

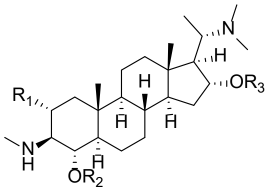

	R_1	R_2	R_3	pred. pIC_{50} (*Pf*)
new_AS_07	H	Bz	Bz	6.6672
new_AS_08	H	Bz(OMe)3	Bz(OMe)3	6.9252
new_AS_09	OH	Bz	Bz	6.7927
new_AS_10	OH	Bz(OMe)3	Bz(OMe)3	7.0541

Bz = Benzoyl 3,4,5-Trimethoxybenzoyl
Bz(OMe)3

3. Conanine derivatives based on compound **5-1** with additional aryl ester moieties at C-4 and C-16 and with/without OH substitution at C-2

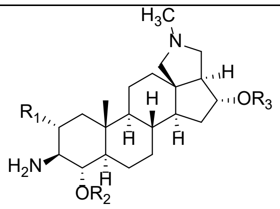

	R_1	R_2	R_3	pred. pIC_{50} (*Pf*)
new_AS_11	H	Bz	Bz	6.6994
new_AS_12	H	Bz(OMe)3	Bz(OMe)3	6.8955
new_AS_13	OH	Bz	Bz	6.8587
new_AS_14	OH	Bz(OMe)3	Bz(OMe)3	7.0549

Bz = Benzoyl 3,4,5-Trimethoxybenzoyl
Bz(OMe)3
